# Autonomous Solar Metallurgy for High‐Purity Gold Recovery from Electronic Waste

**DOI:** 10.1002/advs.77596

**Published:** 2026-09-06

**Authors:** Chaopeng Liu, Jiajun Li, Jialin Ruan, Wei Zhang, Wei Sun, Hongmei Li, Lei Xie, Min Liu

**Affiliations:** ^1^ School of Minerals Processing and Bioengineering Central South University Changsha China; ^2^ Furfure Resources Interface Science and Intelligent Application Research Center (FRISIARC) Changsha China; ^3^ Henan Key Laboratory of Water Pollution Control and Rehabilitation Technology Henan University of Urban Construction Pingdingshan China; ^4^ Hunan Joint International Research Center for Carbon Dioxide Resource Utilization, School of Physics Central South University Changsha China

**Keywords:** bioinspired materials, E‐waste leachates, gold recovery, photothermal‐photochemical coupling, urban mining

## Abstract

Sustainable recovery of precious metals from electronic waste is constrained by the difficulty of integrating rapid capture, complete reduction, and product separation without external reagents or energy‐intensive inputs. Here, we report a bioinspired, defect‐encoded solar metallurgical platform based on copper sulfide (Cu_31_S_16_) that couples light harvesting, photothermal conversion, and intrinsic redox functionality. Copper‐vacancy‐induced mid‐gap states enhance broadband solar absorption, localized thermal amplification, and photoexcited charge generation, while soft sulfide coordination sites selectively bind Au(III). This co‐localized photothermal‐photochemical coupling accelerates interfacial transport and complete multielectron reduction to Au(0), and uniquely triggers a light‐sustained nucleation‐growth process that culminates in spontaneous self‐abscission of millimetre‐scale, high‐purity gold. The integrated mechanism overcomes site‐saturation, delivering an ultrahigh uptake capacity of 6274 mg g^−1^, near‐instantaneous kinetics (>95% removal within 15 s), near‐unity selectivity (K_d_ = 2.5 × 10^7^ mL g^−1^), and wide pH range operation. Continuous‐flow processing of authentic leachates sustains gold recovery for 65 h and yields ∼24 K gold, demonstrating techno‐economic viability with ∼95% reductions in energy, chemical, and carbon footprints relative to conventional routes. By unifying energy conversion, reaction, and separation into one solar‐driven process, this work establishes a self‐powered, self‐separating metallurgical paradigm for low‐carbon recovery of precious and critical metals.

## Graphical abstract

Inspired by microbial strategies for metal uptake and biomineralization, defect‐rich copper sulfide (Cu_31_S_16_) functions as a solar metallurgical platform that unifies Au(III) capture, reduction, and self‐separation. The bioinspired design enables ultrafast gold recovery from electronic‐waste leachates and the spontaneous formation of millimeter‐scale 24 K gold under sunlight.

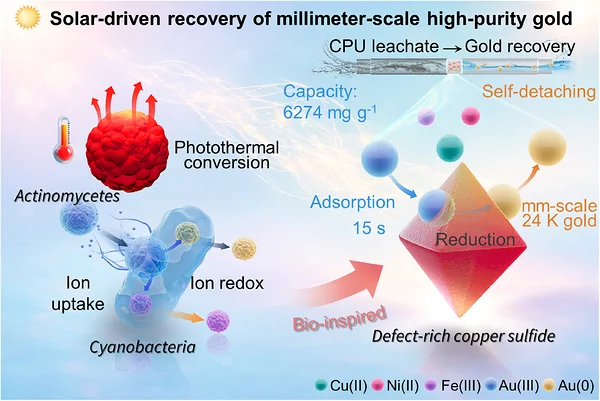

## 1 Introduction

The selective recovery of precious metals from complex waste streams represents a central challenge in sustainable chemistry and circular resource utilization [1, 2]. Gold, a critical material for electronics, catalysis, and emerging clean‐energy technologies, is increasingly sourced from electronic waste (e‐waste), where its concentration often exceeds that of natural ores by one to two orders of magnitude [3, 4, 5]. Despite this opportunity, existing recovery technologies remain energy‐intensive and chemically demanding, typically requiring strong reductants, high temperatures, or multistep purification processes [6]. These constraints arise from a fundamental challenge that efficient recovery requires the simultaneous integration of selective capture, complete reduction, and high‐purity product separation, processes that are traditionally decoupled and energy‐intensive [7, 8, 9]. In conventional systems, these functions remain spatially and operationally disconnected, resulting in energy penalties, chemical consumption, and material losses [10, 11].

Adsorption‐based strategies offer a promising route due to their simplicity and scalability [12, 13, 14, 15]. However, conventional adsorbents are inherently limited by three factors: (i) site‐saturation‐limited capacity [16, 17], (ii) slow interfacial mass transport [12, 13], and (iii) incomplete reduction of Au(III), often yielding Au(I) intermediates that compromise product purity [2, 18]. To overcome these limitations, external reductants or thermal inputs are typically introduced, increasing both energy consumption and environmental impact [19, 20]. These challenges reveal the need for a new metallurgical paradigm that unifies capture, conversion, and separation within a single energy‐efficient process.

**FIGURE 1:**
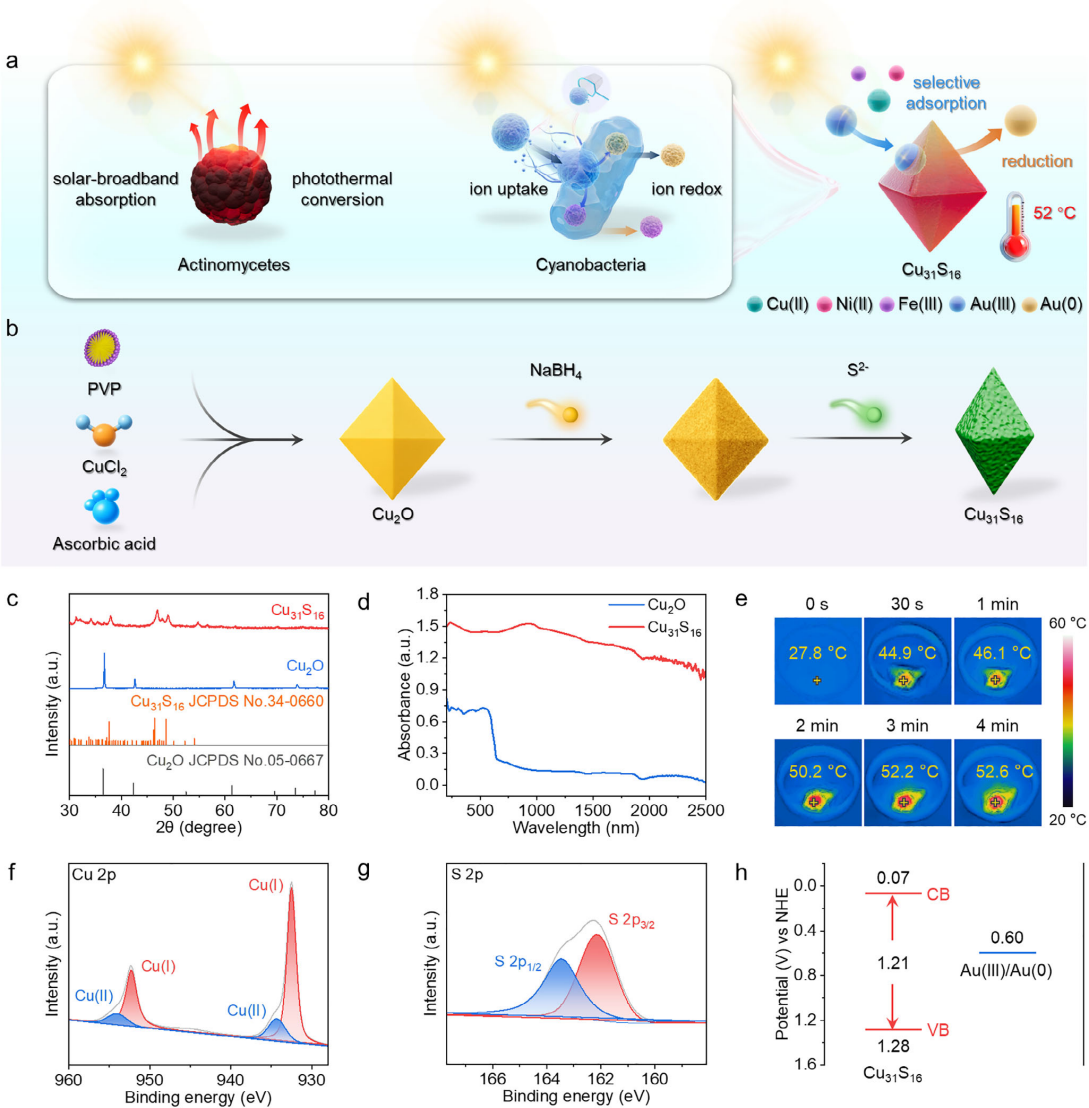
Bioinspired design principle, synthesis strategy, and physicochemical characteristics of the solar‐driven photothermal gold adsorbent. (a) Schematic illustration of the bioinspired concept: broadband solar harvesting generates localized photothermal heating and photoexcited electrons, which synergistically accelerate Au(III) capture and reduction by integrating light absorption, interfacial heat localization, and reactive sulfide binding sites within a single scaffold. (b) Synthetic route for transforming Cu_2_O templates into defect‐rich Cu_31_S_16_ via controlled sulfidation and vacancy engineering. (c) XRD patterns of Cu_2_O and Cu_31_S_16_. (d) UV–vis‐NIR diffuse reflectance spectra of Cu_2_O and Cu_31_S_16_. (e) Infrared thermal images of the Cu_31_S_16_ surface as a function of time under 1‐sun irradiation. (f, g) High‐resolution XPS spectra of Cu 2p (f) and S 2p (g) in Cu_31_S_16_. (h) Energy band alignment of Cu_31_S_16_ relative to the Au(III)/Au(0) redox potential.

Nature offers a compelling blueprint for such integration. Biological systems routinely co‐localize light harvesting, thermal regulation, and redox chemistry to achieve highly efficient energy conversion and chemical transformation (Figure 1a). For example, melanin‐rich microorganisms (e.g., Cryptococcus and actinomycetes) convert broadband solar radiation into localized heat [21], while photosynthetic systems (e.g., cyanobacteria and purple non‐sulfur bacteria) couple photon‐driven charge separation with catalytic reactions [22, 23, 24]. This biological logic points to an attractive design principle for gold recovery, in which photon absorption, heat generation, ion enrichment, and electron transfer are confined to the same reaction interface [25, 26]. Translating this principle into synthetic systems, however, remains a major challenge.

Here, we introduce a bioinspired defect‐engineered solar metallurgical strategy that leverages vacancy‐induced electronic states to integrate photon absorption, photothermal conversion, and redox functionality within a single material platform (Figure 1a). Specifically, copper‐deficient Cu_31_S_16_ is designed to host abundant mid‐gap states that enable broadband solar harvesting and efficient energy conversion. Simultaneously, sulfide ligands act as soft Lewis base sites to selectively bind Au(III), while mixed‐valence Cu(I)/Cu(II) centers provide intrinsic redox activity. Most existing strategies enhance Au(III) recovery through functional group engineering, porous adsorption sites, or externally supplied reducing agents [12, 20]. In contrast, vacancy‐rich Cu_31_S_16_ integrates sulfide‐mediated Au affinity, broadband solar harvesting, photothermal heating, and photoinduced interfacial reduction within a single crystalline material, thereby coupling Au capture, reduction, and metallic growth within a continuous light‐driven process. This architecture creates reactive photothermal‐photochemical microdomains, where light, heat, and electron transfer are co‐localized at the adsorption interface. This design enables three key advances. First, coupled photothermal and photochemical effects dramatically accelerate Au(III) transport and reduction kinetics. Second, intrinsic redox functionality drives complete Au(III)‐to‐Au(0) conversion without external reagents. Third, and most critically, the system evolves from a static adsorbent into a dynamic capture‐reduction‐growth continuum, culminating in spontaneous self‐abscission of high‐purity metallic gold. This emergent behavior effectively integrates reaction and separation, overcoming the intrinsic limitations of conventional adsorption systems. By establishing a direct link between defect‐mediated energy coupling, interfacial reaction dynamics, and macroscopic product separation, this work advances a new conceptual framework for solar‐driven metallurgy. Beyond gold recovery, this strategy provides a generalizable pathway for the sustainable extraction and recycling of critical metals in a low‐carbon circular economy.

## 2 Results and Discussion

### 2.1 Defect‐Engineered Solar Metallurgical Platform

To operationalize the concept of autonomous solar metallurgy, we designed a defect‐engineered photothermal‐redox material that integrates light harvesting, thermal amplification, and metal conversion within a single scaffold. A top‐down sulfidation strategy was developed to transform monodisperse Cu_2_O microcrystals into a defect‐rich Cu_31_S_16_ architecture (Figure 1b). Uniform octahedral Cu_2_O particles (∼1.5 µm, Figure S1) were first prepared via precipitation‐reduction, followed by NaBH_4_ etching and controlled sulfidation with Na_2_S. This transformation proceeds via a Kirkendall‐type diffusion process [27], generating a porous Cu_31_S_16_ architecture enriched with lattice defects (Figure S2).

X‐ray diffraction (XRD) confirmed complete phase conversion from cubic Cu_2_O (JCPDS No. 05–0667) to monoclinic Cu_31_S_16_ (JCPDS No. 34–0660) (Figure 1c). The sulfidation process more than doubled the specific surface area and generated an interconnected microporous framework (Figures S3 and S4), consistent with vacancy‐driven anion exchange [28]. Rietveld refinement revealed significant lattice strain and peak broadening, indicative of abundant copper vacancies [29]. The vacancies are not merely structural defects but function as electronically active centers, introducing mid‐gap states that extend light absorption and stabilize mixed‐valence Cu(I)/Cu(II) species. This defect‐enabled electronic structure establishes the foundation for simultaneous photon capture and redox activation, distinguishing the system from conventional passive adsorbents [30, 31].

Optically, Cu_31_S_16_ exhibited near‐black broadband absorption across the solar spectrum, in stark contrast to Cu_2_O (Figure 1d and Figure S5). Bandgap narrowing derived from Kubelka–Munk analysis confirmed the emergence of vacancy‐induced electronic states that promote nonradiative relaxation and efficient photothermal conversion (Figure S6) [32]. Under simulated solar irradiation, the material exhibited a rapid temperature rise of 17.1°C within 30 s and reaches 52.6°C within 4 min, outperforming Cu_2_O by more than threefold (Figure 1e and Figures S7 and S8). This localized heating generated microscale thermal gradients that directly accelerate interfacial transport and reaction kinetics.

X‐ray photoelectron spectroscopy (XPS) analysis further revealed a mixed‐valence Cu(I)/Cu(II) electronic structure (Cu(I):Cu(II) ≈ 3.98:1; Figure 1f), providing an intrinsic redox reservoir for electron transfer [33, 34]. The S 2p spectrum confirmed abundant sulfide coordination sites (Figure 1g), which act as soft Lewis bases for selective Au(III) binding. Mott–Schottky measurement identified Cu_31_S_16_ as a p‐type semiconductor (Figure S9), with a conduction band position (0.07 V vs. NHE) thermodynamically favorable for Au(III) reduction (Figure 1h) [35]. This band alignment thermodynamically permits direct photoreduction of Au(III). Electrochemical impedance spectroscopy (EIS) and photocurrent measurements further revealed low charge‐transfer resistance and efficient photoinduced carrier mobility (Figures S10 and S11). Therefore, these results demonstrate that Cu_31_S_16_ functions as an integrated solar metallurgical microreactor, in which defect‐mediated light absorption, localized heating and redox‐active coordination sites are co‐localized. This architecture enables the coupling of adsorption, energy conversion and metal reduction within a single material, forming the basis of a self‐driven gold recovery process.

### 2.2 Light‐Driven Kinetic Amplification and Capacity Breakthrough

**FIGURE 2:**
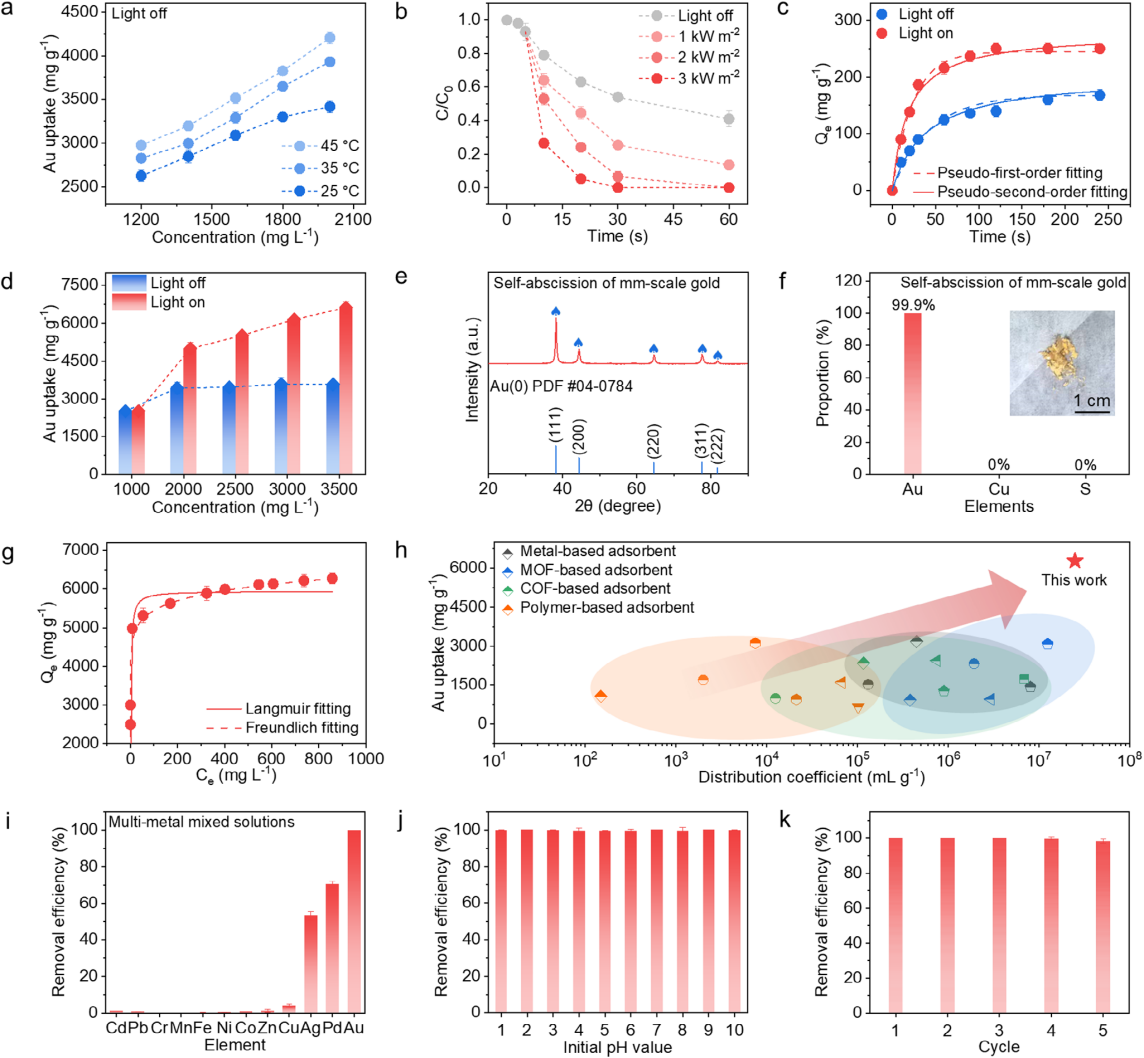
Solar‐enhanced Au(III) capture, reduction and performance benchmarking of Cu_31_S_16_. (a) Effect of temperature on Au(III) adsorption capacity under dark conditions after 12 h. (b) Effect of light intensity on Au(III) adsorption efficiency (50 mg L^−1^) following 5 s of initial dark equilibration. (c) Adsorption kinetics of Au(III) (100 mg L^−1^) under dark and light irradiation. (d) Au(III) adsorption capacity at elevated initial concentrations under dark and light conditions after 12 h. (e) XRD pattern of spontaneously formed millimeter‐scale metallic gold recovered after solar‐driven reduction. (f) ICP analysis of spontaneously formed metallic gold. (g) Au(III) adsorption isotherms under light irradiation after 12 h. (h) Benchmark comparison of maximum Au(III) adsorption capacity and distribution coefficient (K_d_, in mL g^−1^) of Cu_31_S_16_ with representative Au(III) adsorbents. K_d_ quantifies the partitioning of Au(III) between the solid and liquid phases. (i) Au(III) removal efficiency in multi‐metal systems (competing ions: 100 mg L^−1^; Au(III): 10 mg L^−1^). (j) pH‐dependent Au(III) (100 mg L^−1^) removal performance across a wide range. (k) Cycling stability of Cu_31_S_16_ over five adsorption‐desorption cycles.

Thermodynamic analysis under dark conditions revealed that Au(III) adsorption on Cu_31_S_16_ is spontaneous (ΔG° < 0) and endothermic (ΔH° > 0) (Figure 2a and Figure S12 and Table S1), indicating that elevated temperature can enhance uptake. Au(III) removal was subsequently compared under dark and illuminated conditions while maintaining the bulk solution at 25°C. At an initial Au(III) concentration of 50 mg L^−1^, illumination increased the removal efficiency from approximately 60% under dark conditions to nearly 100% after 1 min, together with a corresponding increase in Au uptake (Figures S13 and S14). This pronounced enhancement cannot be attributed solely to bulk‐solution heating and instead indicates a light‐triggered acceleration of the interfacial capture process. This response was further verified by a light‐switching experiment. Only approximately 7% of Au(III) was removed during the initial 5 s in the dark. Upon illumination, the removal efficiency increased sharply, exceeding 55% after a total reaction time of 15 s under one‐sun irradiation and approaching 95% under three‐sun irradiation (Figure 2b). At 100 mg L^−1^, >99% removal was achieved within 2 min under illumination, compared to only 67% after >6 min in the dark (Figure 2c). Kinetic modeling followed a pseudo‐second‐order regime (R^2^ = 0.958–0.974), indicating chemisorption‐controlled uptake (Table S2). However, the key distinction lies in the light‐triggered kinetic amplification: photothermal heating enhances Au(III) diffusivity and collision frequency, while defect‐mediated photoelectrons enable rapid reduction of surface‐bound species. These coupled effects eliminate the classical transport and electron‐transfer bottlenecks that limit conventional adsorption systems. In addition, Cu release was observed during the adsorption process, with a Cu‐release/Au‐recovery mass ratio of ∼3.8% (Figure S15). When the initial Au concentration increased to 500 mg L^−1^, this ratio increased to approximately 4.9% (Figure S16). These results confirm that Au recovery is accompanied by partial Cu dissolution, yet the released Cu mass remains negligible compared to the Au harvested. This behavior is consistent with a redox‐driven process in which AuCl_4_^−^ reduction is coupled to the oxidation and partial extraction of lattice Cu, analogous to galvanic replacement and cation‐exchange processes previously reported for Cu‐containing substrates and Cu‐deficient sulfides [36, 37].

A notable feature is the emergence of a self‐reinforcing reaction pathway. The initial formation of Au(0) nanoparticles introduces localized surface plasmon resonance (LSPR), generating additional hot carriers and heat under illumination. This establishes a positive feedback loop that continuously accelerates both adsorption and reduction [38, 39], effectively transforming the process into a light‐driven autocatalytic system. Vacancy‐poor Cu_2_S was synthesized as a control to further clarify the role of the vacancy‐rich structure (Figure S17). Compared with Cu_2_S, Cu_31_S_16_ exhibited substantially faster Au(III) uptake and a higher adsorption capacity, supporting an important contribution of its vacancy‐rich structure to the enhanced interfacial capture process (Figures S18 and S19). Copper vacancies perturb the local coordination and electronic environment and introduce vacancy‐derived electronic states, thereby creating a more favorable interface for enrichment and subsequent light‐driven interfacial reduction [40, 41].

Illumination fundamentally alters the adsorption thermodynamics. In the dark, capacity plateaued at ∼3418 mg g^−1^ due to site saturation (Figure 2d). Under solar irradiation, capacity increased continuously to 6274 mg g^−1^, a 1.8‐fold enhancement beyond the static adsorption limit. This behavior reflected a transition from static adsorption to a dynamic capture‐reduction‐growth mechanism [42, 43]. Photoreduced Au(0) acted as nucleation seeds, enabling continuous gold accumulation beyond surface binding constraints. Strikingly, accumulated gold evolves into millimetre‐scale metallic particles that spontaneously detach from the surface (Movies S1 and S2). XRD and ICP‐OES confirmed >99.9% purity (∼24 K gold) (Figure 2e,f). Freundlich isotherm fitting (R^2^ = 0.993) outperformed Langmuir modeling (R^2^ = 0.887), indicating heterogeneous multilayer adsorption coupled with nucleation‐growth behavior (Figure 2g and Table S3). This self‐abscission phenomenon eliminates the need for chemical elution or post‐processing, representing a critical step toward process simplification and sustainability.

Cu_31_S_16_ also demonstrated exceptional selectivity in complex systems. In multicomponent solutions, Au(III) removal remains >99.9%, while competing ions show negligible uptake (Figure 2i and Figure S20, and Tables S4 and S5). Although Ag(I) and Pd(II) do interact with the sulfur‐rich surface, Cu_31_S_16_ retains a distinct preference for Au(III). This selectivity is thermodynamically rationalized by the higher standard reduction potential of the AuCl_4_^−^/Au couple (E° = 1.00 V vs. SHE) relative to those of PdCl_4_^2−^/Pd (E° = 0.95 V) and (Ac) Ag/Ag (E° = 0.64 V) [44]. This thermodynamic driving force, combined with strong Au‐S interactions and the rapid nucleation and growth of metallic Au, likely contributes to the observed preferential uptake [45, 46]. The distribution coefficient (K_d_) reached 2.5×10^7^ mL g^−1^, exceeding competing ions by 5–6 orders of magnitude (5.5–110.0 mL g^−1^, Table S5). This performance was attributed to the synergy between soft‐soft coordination (S^2−^‐Au^3+^) and rapid reduction kinetics, which kinetically excludes competing species. The adsorption capacity and K_d_ value of Cu_31_S_16_ were compared with a wide range of reported Au(III) adsorbents, including metal‐, MOF‐, COF‐, polymer‐, and metal‐derived materials (Table S6). As shown in Figure 2h, Cu_31_S_16_ resided in the top‐performing region of the adsorption capacity versus K_d_ value relationship. The adsorption capacity of Cu_31_S_16_ was 2 to 10 times higher than that of reported adsorbents (657–3180 mg g^−1^), and its K_d_ value surpassed all these materials, revealing the combined advantage of ultrahigh capacity and exceptional selectivity of Cu_31_S_16_. Importantly, Cu_31_S_16_ maintained its Au‐removal performance over a broad pH range of 1–10 and during repeated adsorption‐desorption cycles, without detectable structural degradation (Figure 2j,k and Figure S21), demonstrating strong chemical and operational robustness. Its capacity for sustained Au capture without intermediate regeneration was further evaluated by repeatedly exposing Au‐loaded Cu_31_S_16_ to fresh Au(III) solutions (Figure S22). The removal efficiency remained above 99% over ten sequential runs, indicating that progressive Au deposition did not compromise the accessibility or reactivity of the capture interface. Moreover, Cu_31_S_16_ maintained efficient Au(III) capture under acidic conditions and retained its crystallinity and morphology (Figures S23–S26).

### 2.3 Autonomous Capture‐Reduction‐Separation Mechanism

**FIGURE 3:**
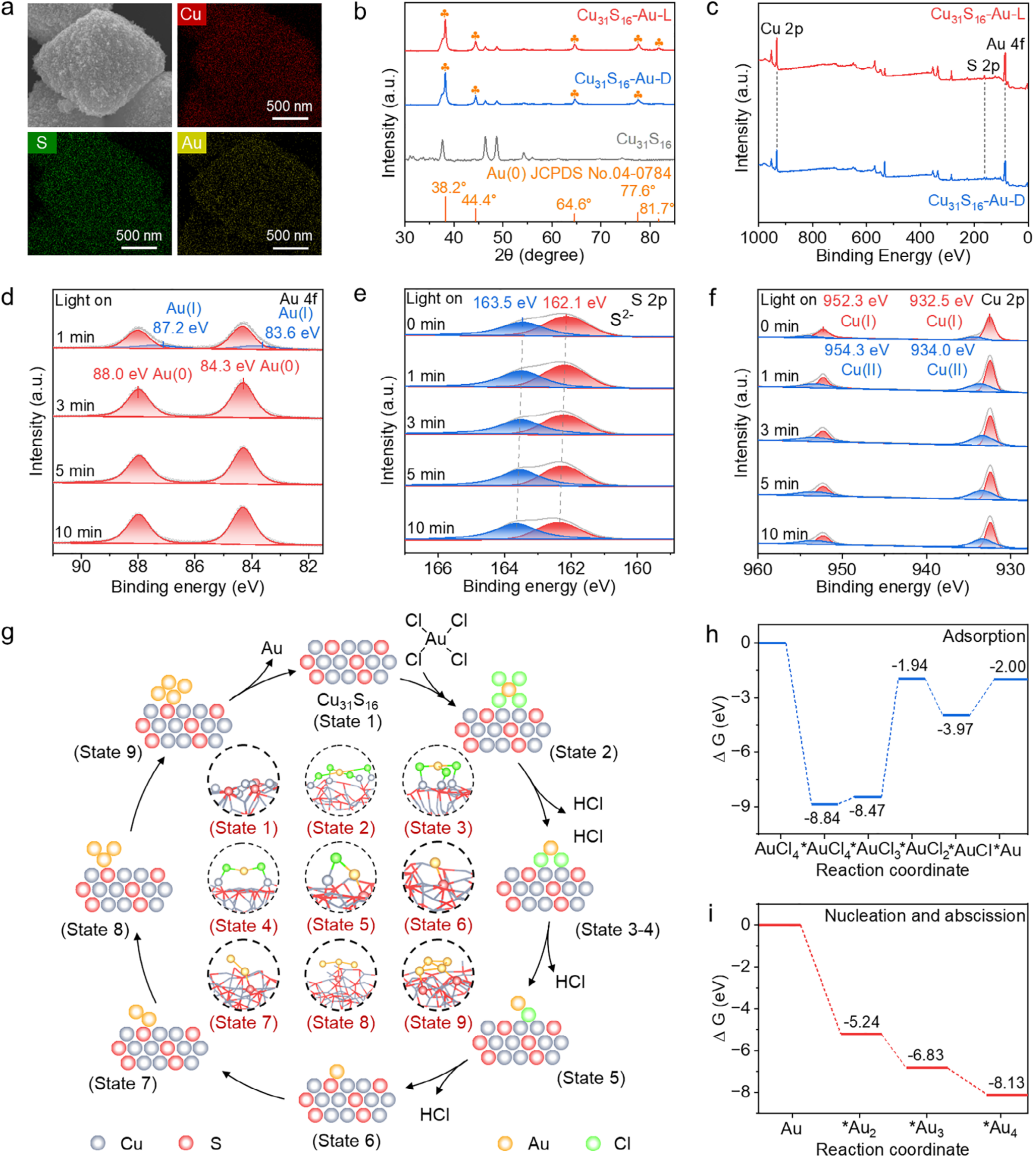
Mechanistic elucidation of solar‐driven Au(III) adsorption‐reduction on Cu_31_S_16_. (a) SEM image and corresponding EDS elemental maps of Cu_31_S_16_ after Au(III) capture under light irradiation. (b) XRD patterns of Cu_31_S_16_ after Au(III) adsorption under dark (Cu_31_S_16_‐Au‐D) and light irradiation (Cu_31_S_16_‐Au‐L) conditions. (c) XPS survey spectra of Cu_31_S_16_‐Au‐D and Cu_31_S_16_‐Au‐L after Au(III) adsorption. (d–f) Time‐resolved XPS evolution of Au 4f (d), S 2p (e), and Cu 2p (f) spectra of Cu_31_S_16_ under light irradiation. (g) Schematic illustration of Au(III) coordination, nucleation, and growth into metallic gold on Cu_31_S_16_. (h) Spin‐polarized DFT‐calculated energy profiles for multinuclear Au(III) adsorption on Cu_31_S_16_. (i) Spin‐polarized DFT‐calculated energy evolution during nucleation, growth, and desorption of gold clusters on Cu_31_S_16_.

To elucidate the origin of the autonomous gold recovery behavior, we tracked the structural and electronic evolution of Cu_31_S_16_ during operation under dark and illuminated conditions. Scanning electron microscopy (SEM) revealed the rapid emergence of uniformly dispersed nanoparticles on the Cu_31_S_16_ surface after Au(III) exposure (Figure 3a), while energy‐dispersive X‐ray spectroscopy (EDS) confirmed the co‐localization of Au with Cu and S, indicating in situ nucleation of gold species during adsorption. XRD verified the formation of crystalline metallic Au(0), with characteristic reflections at 38.2°, 44.4°, 64.6°, 77.6°, and 81.7° (JCPDS No. 04–0784) under both dark (Cu_31_S_16_‐Au‐D) and illuminated (Cu_31_S_16_‐Au‐L) conditions (Figure 3b). Notably, Au(0) peak intensities were substantially higher under illumination, evidencing a light‐accelerated and more complete Au(III)‐to‐Au(0) reduction pathway.

XPS provided direct insights into the reduction intermediates (Figure 3c). Under dark conditions, both Au(I) (87.4, 83.8 eV) and Au(0) (88.3, 84.5 eV) species were detected, indicating a kinetically limited multistep reduction pathway in which Au(I) accumulates as a metastable intermediate (Figure S27). Concurrent oxidation of Cu(I) to Cu(II) confirms that lattice Cu acts as the primary electron donor (Figure S28), but electron supply was kinetically insufficient to fully overcome the Au(I) to Au(0) barrier in the absence of external energy input. In contrast, illumination eliminated Au(I) intermediates entirely, yielding exclusively metallic Au(0). The enhanced Cu(I)→Cu(II) transition under light confirmed accelerated charge extraction, demonstrating that photoexcited carriers overcome the intrinsic kinetic barrier of the Au(I)→Au(0) step. This enhancement originates from vacancy‐induced mid‐gap states, which enable efficient broadband photon harvesting and generate a coupled flux of heat and charge carriers. Simultaneously, sulfide ligands act as soft Lewis bases, selectively coordinating Au(III) and stabilizing reaction intermediates (Figure S29). The resulting Cu‐S‐Au interfacial motifs function as localized photothermal‐redox microdomains, where energy input (light), reactant concentration (Au species), and electron supply are spatially co‐localized. This integration suppresses intermediate accumulation and enables rapid multielectron reduction. Time‐resolved XPS further revealed the evolution of the captured Au species under illumination. Within 1 min of irradiation, ∼92.6% of surface Au existed as Au(0), increasing to complete conversion within 3 min (Figure 3d). Concurrent shifts in S 2p and the progressive decrease in the Cu(I)/Cu(II) ratio (Figure 3e,f) further supported that sulfide sites are chiefly responsible for Au‑species capture, interfacial accumulation, and stabilization of reaction intermediates, whereas Cu(I) sites function as the predominant electron source for the reduction of Au(III). These results demonstrated that photothermal heating (enhancing mass transport and reaction frequency) and photochemical charge injection (lowering activation barriers) operate synergistically, establishing a self‐sustaining reaction environment under solar input.

Spin‐polarized density functional theory (DFT) calculations revealed the atomistic pathway underlying this behavior. Au(III) adsorption initiated with Cu‐assisted chloride abstraction, weakening Au─Cl bonds and facilitating stepwise dechlorination (Figure 3g,h). The exergonic nature of these steps explained the experimentally observed Au(I) intermediates. As dechlorination proceeds, Au‐S coordination dominates, stabilizing reduced species and enabling the formation of Au─Au bonds. At this stage, the reaction transitions from surface‐limited chemistry to nucleation‐driven metallic growth, where Au‐Au cohesion becomes energetically favored over Au‐surface interactions (Figure 3i). Consistently, the binding energies of *Au_2_, *Au_3_, and *Au_4_ become progressively more negative, indicating increasing thermodynamic stabilization as the Au clusters grow. Once nucleation occurs, the system evolves into a dynamic capture‐reduction‐growth continuum, in which newly formed Au clusters act as energetically favorable growth centers for the subsequent reduction and deposition of Au species. The increasing stability of larger Au clusters supports a positive‐feedback pathway in which the initially deposited Au(0) promotes continued Au nucleation and growth, consistent with previously reported seed‐mediated Au growth mechanisms [47, 48]. As clusters grow beyond a critical size, interfacial adhesion weakens relative to cohesive forces. Under photothermal heating and hydrodynamic perturbation, this imbalance drives the spontaneous detachment of metallic gold. This self‐abscission mechanism enables direct physical separation of high‐purity gold without chemical stripping, representing a key step toward process simplification.

Overall, the mechanism can be described as an autonomous solar metallurgical cycle: (i) selective coordination of Au(III), (ii) light‐driven electron and heat generation, (iii) rapid multielectron reduction, (iv) nucleation and growth of metallic Au, and (v) spontaneous product separation. Unlike conventional photocatalytic systems, in which illumination primarily drives metal‐ion reduction, the initially formed Au nuclei promote further Au deposition and growth, ultimately producing millimetre‐scale metallic particles that spontaneously detach from the material surface. By continuously coupling ion recognition, reduction, metallic growth, and physical separation, the overall process provides an integrated metallurgical cycle, underpinning its high efficiency, process simplicity, and sustainability.

### 2.4 Scaling up Gold Recovery from Authentic E‐Waste Leachates

To evaluate practical applicability, the system was tested using authentic leachates derived from discarded central processing unit (CPU) pins. Four commonly used leaching systems, namely aqua regia, thiosulfate, trichloroisocyanuric acid (TCCA), and N‐bromosuccinimide/pyridine (NBS/Py), were employed to extract gold from the CPU pins (Methods 4). Despite the presence of high concentrations of competing metals such as Cu(II) and Ni(II), Cu_31_S_16_ maintained >99.6% Au recovery from the aqua regia, TCCA, and NBS/Py leachates (Figures S30–S32), confirming that the selective coordination‐reduction mechanism remains operative under realistic, high‐ionic‐strength conditions. These base metals should not be regarded merely as disposable contaminants; after appropriate solution conditioning, Cu and Ni can be recovered as secondary resources through established solvent‐extraction, precipitation, and electrowinning processes [49]. In the thiosulfate system, however, Au recovery was significantly lower (32.5%) (Figure S33), consistent with the substantially lower reduction potential of the Au(S_2_O_3_)_2_^3−^/Au couple (0.15 V vs SHE) compared with that of AuCl_4_^−^/Au couple (1.00 V versus SHE) [50, 51]. This lower potential, together with the strong stabilization of Au(I) by thiosulfate, likely weakens the thermodynamic driving force for interfacial Au reduction and deposition on Cu_31_S_16_. To further validate the versatility of the system, we extended the evaluation to other authentic e‐waste streams, including mobile phone motherboards. After aqua regia leaching, the resulting leachate was processed using the identical adsorption protocol, achieving >99% Au recovery (Figure S34).

**FIGURE 4:**
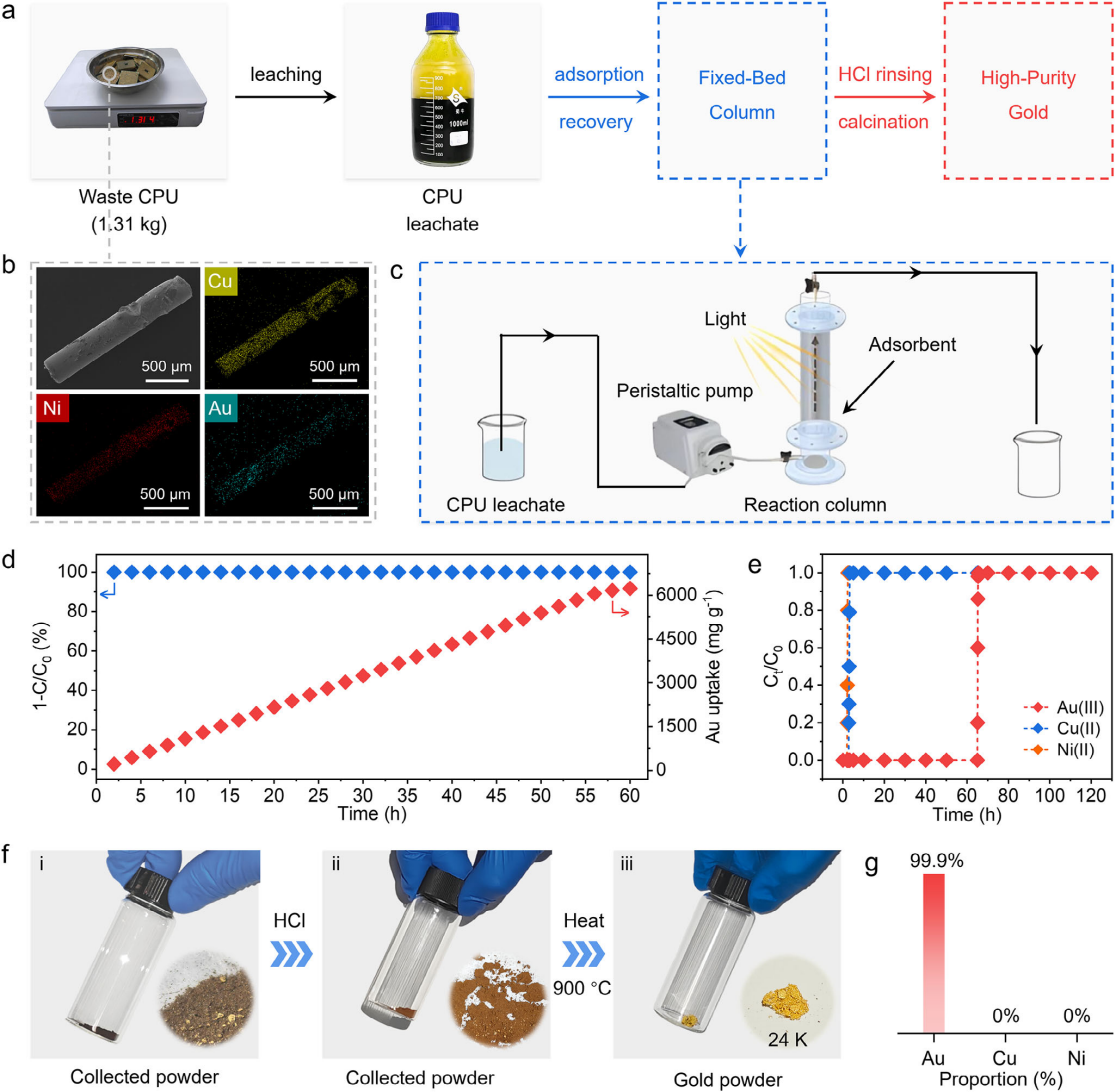
Solar‐driven recovery of high‐purity gold from e‐waste leachate with potential for large‐scale utilization. (a) Technology roadmap of gold extraction based on the Defect‐Encoded Solar Metallurgy system. (b) SEM image and corresponding elemental maps of a representative CPU pin. (c) Schematic illustration of the continuous‐flow fixed‐bed column for solar‐assisted gold recovery from CPU leachate. (d) Continuous‐flow performance during treatment of authentic CPU leachate over 60 h under light irradiation, showing sustained Au removal efficiency (blue) and cumulative adsorption capacity (red). (e) Dynamic breakthrough curves of Au in a Cu_31_S_16_ packed‐bed column for CPU leachate. (f) Macroscopic evolution of recovered gold: visible surface‐bound gold nanoparticles after adsorption (i), acid‐treated intermediate (ii), and millimetre‐scale metallic gold particles after calcination (iii). (g) ICP analysis confirming the recovered gold purity of 99.9% (∼24 K).

A process‐level demonstration was conducted using 1.31 kg of waste CPUs as feedstock (Figure 4a). Mechanical dismantling and separation yielded Au‐containing pins, which were characterized by SEM‐EDS (Figure 4b). Following aqua regia leaching, the resulting solution (173.1 mg L^−1^ Au) was directly treated in a Cu_31_S_16_‐packed fixed‐bed column under solar irradiation (Figure 4c). The system exhibited stable continuous operation for ∼60 h with >98.8% Au removal efficiency (Figure 4d). The cumulative uptake (6230.5 mg g^−1^) closely matches batch performance, demonstrating scalability without loss of efficiency. Breakthrough analysis shows rapid elution of Cu and Ni (<3.2 h), while Au remains strongly retained for >65 h (Figure 4e), corresponding to orders‐of‐magnitude selectivity under dynamic flow conditions. Recovered gold was isolated through post‐treatment, yielding metallic products with >99.9% purity (∼24 K) (Figure 4f,g), meeting standards for direct reuse. Importantly, the system preserved stable performance and structural integrity throughout prolonged operation, underscoring its durability for practical deployment. Based on established photoreactor‐engineering studies, practical implementation could employ shallow flat‐plate or annular reactors, translucent monolithic flow structures, or internally illuminated optical‐fiber/LED modules, while throughput could be increased by numbering up multiple short‐optical‐path channels [52, 53, 54].

From a sustainability perspective, this process introduces several key advantages over conventional hydrometallurgical routes: elimination of external reductants and thermal inputs, direct solid‐phase recovery without solvent‐intensive stripping, compatibility with continuous‐flow operation, and high selectivity reducing downstream purification burden. Therefore, these features enable a transition from energy‐ and chemical‐intensive metallurgy toward a low‐carbon, solar‐driven urban mining platform. The successful kg‐scale demonstration reveals the potential for integration into decentralized recycling systems, offering a viable pathway for closing the loop on precious metal resources.

### 2.5 Environmental and Techno‐Economic Analyses

**FIGURE 5:**
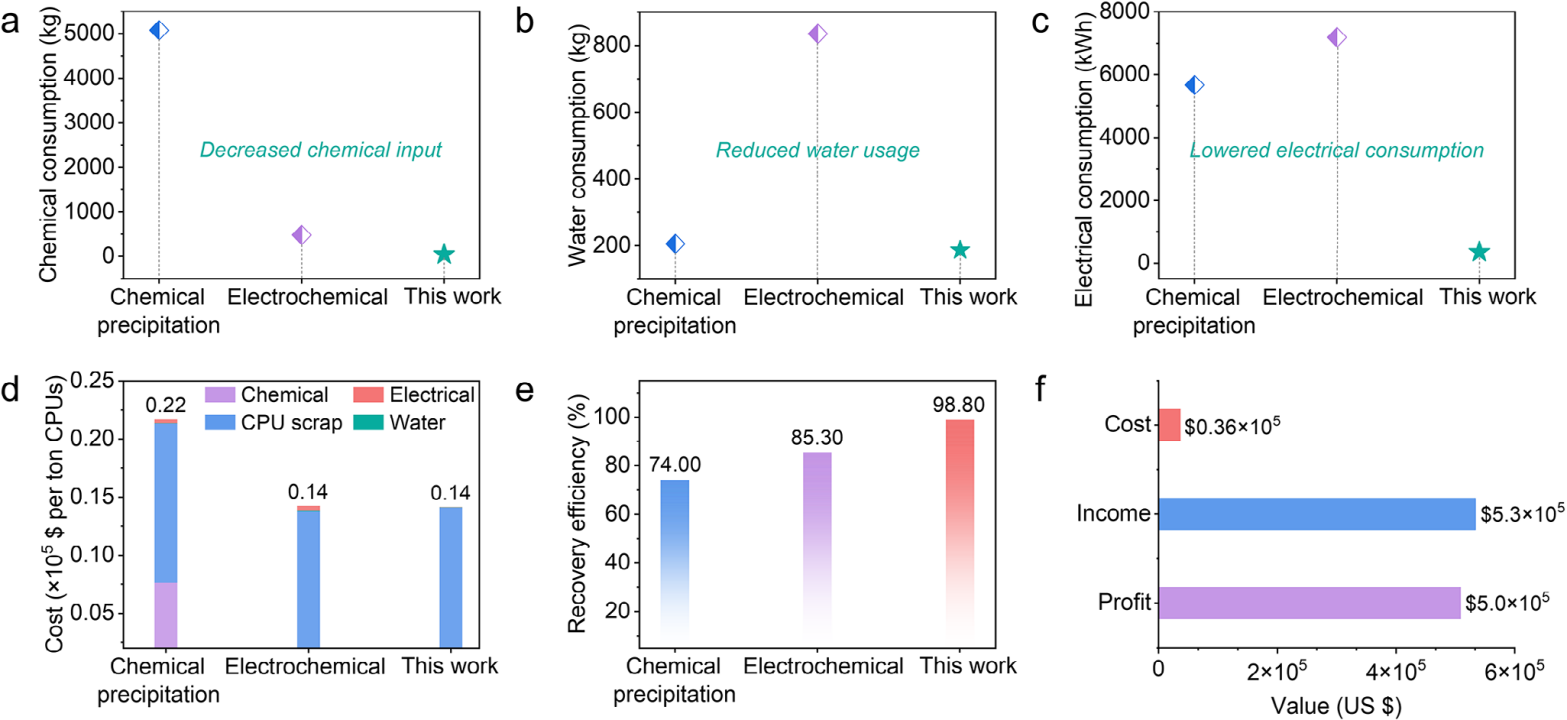
Environmental and techno‐economic performance of gold recovery processes, taking the recovery of 1 tonne of CPU scraps as an example. (a–c) Chemical, water, and electricity consumption of different gold recovery methods. (d) Material and electricity costs analysis of different gold recovery methods. (e) Recovery efficiency comparison of different gold recovery methods. (f) Economic analysis of gold recovery from CPU leachate utilizing the Cu_31_S_16_ adsorbent.

To assess the sustainability advantages of the proposed system, we compared its environmental and techno‐economic performance with those of representative chemical precipitation and electrochemical recovery routes, using a functional unit of 1 tonne of pretreated CPU feedstock (Figure 5a–c and Tables S7–S9). The Cu_31_S_16_‐enabled solar metallurgy process exhibits substantially reduced resource intensity across all key metrics. Electricity consumption is decreased to ∼4.9% of that required for electrochemical recovery (7,187 kWh t^−1^), reflecting the replacement of external electrical input with direct solar energy utilization. Similarly, chemical consumption is reduced to ∼0.9% of that required for precipitation‐based recovery (5,076 kg t^−1^), owing to the elimination of external reductants and precipitants. Water usage is also minimized due to the absence of repeated washing and stripping steps. Beyond resource efficiency, the process fundamentally alters the energy‐material coupling in metallurgical operations, shifting from externally driven inputs to intrinsic, solar‐powered conversion within the material itself. This transition enables a reduction in both operational complexity and indirect emissions, suggesting the potential for decentralized and low‐carbon deployment in urban mining systems.

Recent studies have established complementary routes for sustainable gold processing, including photocatalytic leaching, light‐driven Au extraction from end‐of‐life electronics, and TCCA‐based leaching coupled with polymer sorption [2, 6, 55]. These works provide useful benchmarks for techno‐economic analysis (TEA), which focuses on downstream Au recovery [56]. Specifically, this analysis covers CPU feedstock leaching (HCl and HNO_3_ consumption), Cu_31_S_16_ synthesis, and gold capture, separation, and purification stages. Across all routes, feedstock procurement dominates total costs (63.3–97.0%), reflecting the intrinsic variability of e‐waste composition and market pricing (Figure 5d). While electrochemical recovery is characterized by high electricity demand and precipitation by substantial reagent consumption, the proposed approach minimizes both contributions, resulting in a cost structure largely independent of energy and chemical inputs. Due to its higher recovery efficiency, the solar metallurgical system yields 1.33‐ and 1.16‐fold greater gold recovery than precipitation and electrochemical routes, respectively (Figure 5e), corresponding to an estimated revenue of ∼US$0.50 million per ton of processed feedstock (Figure 5f). Notably, the economic advantage becomes more pronounced under scenarios of rising energy or reagent costs, underscoring the resilience of the approach to market fluctuations. Therefore, the defect‐encoded solar metallurgy strategy not only reduces environmental impacts but also decouples economic performance from energy and chemical price volatility, providing a scalable pathway toward sustainable and economically robust precious metal recovery.

## 3 Conclusions

This work establishes an autonomous solar metallurgical paradigm in which energy capture, chemical transformation, and product separation are intrinsically integrated within a single material system. The defect‐engineered Cu_31_S_16_ simultaneously enables broadband solar absorption, efficient photothermal conversion, selective Au(III) coordination, and intrinsic redox activity. Under illumination, these coupled functions generate localized heat and photoexcited carriers that accelerate interfacial transport and drive complete multielectron reduction. Mechanistically, Au(III) undergoes Cu‐assisted dechlorination followed by light‐enhanced reduction, while sulfide coordination sites stabilize intermediates and direct nucleation. The resulting Au(0) seeds initiate a self‐sustaining nucleation‐growth cycle, overcoming the site‐limited constraints of conventional adsorbents and enabling an ultrahigh capacity of 6274 mg g^−1^. Crucially, continued growth leads to the spontaneous self‐abscission of millimetre‐scale, high‐purity gold, enabling direct product recovery without chemical stripping or additional separation steps.

At the process level, the system demonstrates exceptional selectivity, broad pH tolerance, and stable long‐term operation in continuous‐flow recovery from authentic e‐waste streams at kilogram scale, revealing its readiness for practical deployment. Environmental and techno‐economic analyses confirm minimal energy and chemical inputs alongside strong economic returns, positioning the technology as a viable alternative to conventional metallurgical processes. More broadly, this study introduces a capture‐conversion‐separation framework driven by defect‐mediated energy coupling, redefining how solar energy can be harnessed in resource recovery. By transforming metallurgy from an externally driven, energy‐intensive process into a self‐sustained, low‐carbon system, this approach provides a generalizable strategy for extracting valuable metals from complex waste streams. Extending this concept to other materials and target elements could accelerate the transition toward a circular, decentralized, and climate‐compatible resource economy.

## Author Contributions

**Chaopeng Liu**: conceptualization, methodology, investigation, visualization, Writing – original draft, Writing – review and editing. **Jiajun Li**: methodology, investigation. **Jialin Ruan**: visualization. **Wei Zhang**: investigation. **Wei Sun**: methodology, conceptualization. **Lei Xie**: supervision, writing – review and editing, writing – original draft, conceptualization, methodology. **Hongmei Li**: conceptualization, methodology. **Min Liu**: supervision, writing – review and editing, writing – original draft, conceptualization, methodology.

## Funding

The National Key R&D Program of China (2024YFC3712104 and 2023YFC2908900), the Science and Technology Innovation Leading Talent Program of Hunan Province (2024RC1018 and 2023RC1012), National Natural Science Foundation of China (22376222), Natural Science Foundation of Hunan Province (2026JJ3004), Fundamental Research Funds For the Central Universities of Central South University (2026ZZTS0043), Central South University Research Program of Advanced Interdisciplinary Studies (2023QYJC012) and the Special Fund of Henan Key Laboratory of Water Pollution Control and Rehabilitation Technology (CJSZ2025003).

## Conflicts of Interest

The authors declare no conflicts of interest.

## Supporting information




**Supporting File 1**: advs77596‐sup‐0001‐SuppMat.docx.

**Supporting File 2**: advs77596‐sup‐0002‐MovieS1.mp4.

**Supporting File 3**: advs77596‐sup‐0003‐MovieS2.mp4.

## Data Availability

The data that supports the findings of this study are available in the supplementary material of this article.

## References

[1] Y. Chen , Q. Qiao , J. Cao , H. Li , and Z. Bian , “Precious Metal Recovery,” Joule 5, no. 12 (2021): 3097–3115, 10.1016/j.joule.2021.11.002.

[2] Y. Chen , M. Xu , J. Wen , et al., “Selective Recovery of Precious Metals Through Photocatalysis,” Nature Sustainability 4, no. 7 (2021): 618–626, 10.1038/s41893-021-00697-4.

[3] F. Che , J. T. Gray , S. Ha , and J.‐S. McEwen , “Improving Ni Catalysts Using Electric Fields: A DFT and Experimental Study of the Methane Steam Reforming Reaction,” ACS Catalysis 7, no. 1 (2017): 551–562, 10.1021/acscatal.6b02318.

[4] Y. Chen , S. Guan , H. Ge , et al., “Photocatalytic Dissolution of Precious Metals by TiO_2_ through Photogenerated Free Radicals,” Angewandte Chemie International Edition 61, no. 50 (2022): 202213640, 10.1002/anie.202213640. 36282184

[5] X. Chen , S. Guan , J. Zhou , et al., “Photocatalytic Free Radical‐Controlled Synthesis of High‐Performance Single‐Atom Catalysts,” Angewandte Chemie International Edition 62, no. 45 (2023): 202312734, 10.1002/anie.202312734. 37735738

[6] H. Shang , Y. Chen , S. Guan , et al., “Scalable and Selective Gold Recovery from End‐of‐Life Electronics,” Nature Chemical Engineering 1, no. 2 (2024): 170–179, 10.1038/s44286-023-00026-w.

[7] X. Cui , L. Zhang , J. Zhang , et al., “A Novel Metal‐Organic Layered Material with Superior Supercapacitive Performance Through Ultrafast and Reversible Tetraethylammonium Intercalation,” Nano Energy 59 (2019): 102–109, 10.1016/j.nanoen.2019.02.034.

[8] Y. Ren , S. J. Hersch , X. He , R. Zhou , T. G. Dong , and Q. Lu , “A Lightweight, Mechanically Strong, and Shapeable Copper‐Benzenedicarboxylate/Cellulose Aerogel for Dye Degradation and Antibacterial Applications,” Separation and Purification Technology 283 (2022): 120229, 10.1016/j.seppur.2021.120229.

[9] F. Che , J. T. Gray , S. Ha , N. Kruse , S. L. Scott , and J.‐S. McEwen , “Elucidating the Roles of Electric Fields in Catalysis: A Perspective,” ACS Catalysis 8, no. 6 (2018): 5153–5174, 10.1021/acscatal.7b02899.

[10] M. Miyauchi , M. Takashio , and H. Tobimatsu , “Photocatalytic Activity of SrTiO_3_ Codoped with Nitrogen and Lanthanum under Visible Light Illumination,” Langmuir 20, no. 1 (2004): 232–236, 10.1021/la0353125. 15745026

[11] M. Yong , Y. Yang , L. Sun , et al., “Nanofiltration Membranes for Efficient Lithium Extraction From Salt‐Lake Brine: A Critical Review,” ACS Environmental Au 5, no. 1 (2025): 12–34, 10.1021/acsenvironau.4c00061. 39830721 PMC11740921

[12] X. Li , S. Chen , X. Hu , and F. Zi , “Efficient and Selective Recovery of Gold From Thiosulfate Leaching Solution Using Functionalized β‐Cyclodextrins Synthesized by a Steric Hindrance Strategy,” Advanced Functional Materials 34, no. 1 (2024): 2307472, 10.1002/adfm.202307472.

[13] J. Cao , Z. Xu , Y. Chen , et al., “Tailoring the Asymmetric Structure of NH_2_‐UiO‐66 Metal‐Organic Frameworks for Light‐promoted Selective and Efficient Gold Extraction and Separation,” Angewandte Chemie International Edition 62, no. 18 (2023): 202302202, 10.1002/anie.202302202. 36866944

[14] W. Guo , J. Liu , H. Tao , et al., “Covalent Organic Framework Nanoarchitectonics: Recent Advances for Precious Metal Recovery,” Advanced Materials 36, no. 33 (2024): 2405399, 10.1002/adma.202405399. 38896104

[15] L. Chen , J. Tang , X. Zhang , S. Wang , and Z. Ren , “A Novel Benzothiazole Modified Chitosan with Excellent Adsorption Capacity for Au(III) in Aqueous Solutions,” International Journal of Biological Macromolecules 193 (2021): 1918–1926, 10.1016/j.ijbiomac.2021.11.023. 34752796

[16] C. Yue , H. Sun , W.‐J. Liu , et al., “Environmentally Benign, Rapid, and Selective Extraction of Gold From Ores and Waste Electronic Materials,” Angewandte Chemie International Edition 56, no. 32 (2017): 9331–9335, 10.1002/anie.201703412. 28613435

[17] A. S. Varela , “Gold Extracted From Wastewater as an Efficient MOF‐Supported Electrocatalyst,” Angewandte Chemie International Edition 62, no. 12 (2023): 202217395, 10.1002/anie.202217395. 36645803

[18] R. Li , S. Yan , T. Xue , et al., “A MOF/poly(thioctic acid) Composite for Enhanced Gold Extraction from Water Matrices,” Nano Research 17, no. 1 (2024): 382–389, 10.1007/s12274-023-6077-0.

[19] J. L. Ferry , P. Craig , C. Hexel , et al., “Transfer of Gold Nanoparticles from the Water Column to the Estuarine Food Web,” Nature Nanotechnology 4, no. 7 (2009): 441–444, 10.1038/nnano.2009.157. 19581897

[20] R. Sharma , Y. Kim , E. Lee , and H.‐I. Lee , “Ultrapure Gold Recovery from Electronic Waste as Nanoparticles Immobilized in Hydrogel Matrix,” Chemical Engineering Journal 499 (2024): 156539, 10.1016/j.cej.2024.156539.

[21] K.‐L. Wang , X. Ma , D.‐B. Li , et al., “Single Phototrophic Bacterium‐Mediated Iron Cycling in Aquatic Environments,” Research 7 (2024): 0528, 10.34133/research.0528. 39559346 PMC11570789

[22] A. Bose , E. J. Gardel , C. Vidoudez , E. A. Parra , and P. R. Girguis , “Electron Uptake by Iron‐Oxidizing Phototrophic Bacteria,” Nature Communications 5, no. 1 (2014): 3391, 10.1038/ncomms4391. 24569675

[23] J. Neu , C. C. Shipps , M. J. Guberman‐Pfeffer , et al., “Microbial Biofilms as Living Photoconductors Due to Ultrafast Electron Transfer in Cytochrome OmcS Nanowires,” Nature Communications 13, no. 1 (2022): 5150, 10.1038/s41467-022-32659-5. PMC9452534 36071037

[24] S.‐L. Gong , Y. Tian , G.‐P. Sheng , and L.‐J. Tian , “Dual‐Mode Harvest Solar Energy for Photothermal Cu_2‐x_Se Biomineralization and Seawater Desalination by Biotic‐Abiotic Hybrid,” Nature Communications 15, no. 1 (2024): 4365, 10.1038/s41467-024-48660-z. PMC11111681 38778052

[25] X. Cui , Q. Ruan , X. Zhuo , et al., “Photothermal Nanomaterials: A Powerful Light‐to‐Heat Converter,” Chemical Reviews 123, no. 11 (2023): 6891–6952, 10.1021/acs.chemrev.3c00159. 37133878 PMC10273250

[26] J. Cao , Y. Chen , H. Shang , et al., “Aqueous Photocatalytic Recycling of Gold and Palladium from Waste Electronics and Catalysts,” ACS ES&T Engineering 2, no. 8 (2022): 1445, 10.1021/acsestengg.1c00505.

[27] H. Park , J. Kwon , H. Choi , D. Shin , T. Song , and X. W. D. Lou , “Unusual Na^+^ Ion Intercalation/Deintercalation in Metal‐Rich Cu_1.8_S for Na‐Ion Batteries,” ACS Nano 12, no. 3 (2018): 2827–2837, 10.1021/acsnano.8b00118. 29505231

[28] Z.‐J. Qiu , Y. Xing , J. Brugger , et al., “Vacancies in Sulfides Facilitate Fluid‐Induced Solid‐State Diffusion and Critical Metals Accumulation,” Nature Communications 16, no. 1 (2025): 1835, 10.1038/s41467-025-57171-4. PMC11842834 39979275

[29] P. Muhammad , A. Zada , J. Rashid , et al., “Defect Engineering in Nanocatalysts: From Design and Synthesis to Applications,” Advanced Functional Materials 34, no. 29 (2024): 2314686, 10.1002/adfm.202314686.

[30] C.‐P. Lu , G. Li , J. Mao , L.‐M. Wang , and E. Y. Andrei , “Bandgap, Mid‐Gap States, and Gating Effects in MoS_2_ ,” Nano Letters 14, no. 8 (2014): 4628–4633, 10.1021/nl501659n. 25004377

[31] K. Konstantinou , F. C. Mocanu , T.‐H. Lee , and S. R. Elliott , “Revealing the Intrinsic Nature of the Mid‐Gap Defects in Amorphous Ge_2_Sb_2_Te_5_ ,” Nature Communications 10, no. 1 (2019): 3065, 10.1038/s41467-019-10980-w. PMC6624207 31296874

[32] M. Gao , L. Zhu , C. K. Peh , and G. W. Ho , “Solar Absorber Material and System Designs for Photothermal Water Vaporization Towards Clean Water and Energy Production,” Energy & Environmental Science 12, no. 3 (2019): 841–864, 10.1039/C8EE01146J.

[33] W. Zhong , Z. Gong , Z. He , et al., “Modulating Surface Oxygen Species via Facet Engineering for Efficient Conversion of Nitrate to Ammonia,” Journal of Energy Chemistry 78 (2023): 211–221, 10.1016/j.jechem.2022.11.024.

[34] B. Liu , X. Yao , Z. Zhang , et al., “Synthesis of Cu_2_O Nanostructures With Tunable Crystal Facets for Electrochemical CO_2_ Reduction to Alcohols,” ACS Applied Materials & Interfaces 13, no. 33 (2021): 39165–39177, 10.1021/acsami.1c03850. 34382393

[35] N. Zhang , Y. Wang , M. Liu , et al., “Hollow Cu_2‐x_ S@NiFe Layered Double Hydroxide Core–Shell S‐Scheme Heterojunctions With Broad‐Spectrum Response and Enhanced Photothermal‐Photocatalytic Performance,” Small 20, no. 33 (2024): 2400652, 10.1002/smll.202400652. 38552224

[36] A. P. O'Mullane , S. J. Ippolito , A. M. Bond , and S. K. Bhargava , “A Study of Localised Galvanic Replacement of Copper and Silver Films with Gold Using Scanning Electrochemical Microscopy,” Electrochemistry Communications 12, no. 5 (2010): 611–615, 10.1016/j.elecom.2010.02.012.

[37] K. Miszta , G. Gariano , R. Brescia , et al., “Selective Cation Exchange in the Core Region of Cu_2–x_Se/Cu_2–x_S Core/Shell Nanocrystals,” Journal of the American Chemical Society 137, no. 38 (2015): 12195–12198, 10.1021/jacs.5b06379. 26360611 PMC4591528

[38] C. Liu , Z. Wang , S. Zhang , et al., “Effective and Selective Gold Recovery Based on Synergistic Bimetallic‐Hydroxyl Metal–Organic Frameworks,” Chemical Engineering Journal 505 (2025): 159231, 10.1016/j.cej.2025.159231.

[39] C. Liu , Y. Gou , W. Tian , et al., “Leveraging Metal Sites and Hydroxyl Groups in Bimetal‐Organic Framework for Effective Gold Extraction from e‐Waste Leachate,” Chemical Engineering Journal 507 (2025): 160672, 10.1016/j.cej.2025.160672.

[40] S. Li , H. Duan , J. Yu , et al., “Cu Vacancy Induced Product Switching From Formate to CO for CO_2_ Reduction on Copper Sulfide,” ACS Catalysis 12, no. 15 (2022): 9074–9082, 10.1021/acscatal.2c01750.

[41] X. Shi , W. Dai , X. Dong , Q. Ren , J. Sheng , and F. Dong , “Dual Cu and S Vacancies boost CO_2_ Photomethanation on Cu_1.95_S_1‐x_: Vacancy‐Regulated Selective Photocatalysis,” Applied Catalysis B: Environmental 339 (2023): 123147, 10.1016/j.apcatb.2023.123147.

[42] D. Mei and B. Yan , “Rapid Detection and Selective Extraction of Au (III) from Electronic Waste Using an Oxime Functionalized MOF‐on‐MOF Heterostructure,” Small 19, no. 48 (2023): 2304811, 10.1002/smll.202304811. 37507821

[43] T. Zhang , Z. Hou , X. An , et al., “Engineering Hydroxyls on a Metal‐Organic Framework as Adsorbers and High‐Activity Dehydrochlorination Sites for Selective Gold Recovery,” Resources, Conservation and Recycling 199 (2023): 107286, 10.1016/j.resconrec.2023.107286.

[44] Y. Su , A. Berbille , X.‐F. Li , et al., “Reduction of Precious Metal Ions in Aqueous Solutions by Contact‐Electro‐Catalysis,” Nature Communications 15, no. 1 (2024): 4196, 10.1038/s41467-024-48407-w. PMC11101412 38760357

[45] Y. Xue , X. Li , H. Li , and W. Zhang , “Quantifying Thiol–Gold Interactions Towards the Efficient Strength Control,” Nature Communications 5, no. 1 (2014): 4348, 10.1038/ncomms5348. 25000336

[46] J. Luo , J.‐T. Lin , X. Luo , et al., “Cyclic Trinuclear Units Based Covalent Metal‐Organic Frameworks for Gold Recovery,” Angewandte Chemie International Edition 64, no. 27 (2025): 202502749, 10.1002/anie.202502749. 40295201

[47] T.‐H. Yang , S. Zhou , K. D. Gilroy , et al., “Autocatalytic Surface Reduction and its Role in Controlling Seed‐Mediated Growth of Colloidal Metal Nanocrystals,” Proceedings of the National Academy of Sciences 114, no. 52 (2017): 13619–13624, 10.1073/pnas.1713907114. PMC5748196 29229860

[48] N. D. Burrows , S. Harvey , F. A. Idesis , and C. J. Murphy , “Understanding the Seed‐Mediated Growth of Gold Nanorods Through a Fractional Factorial Design of Experiments,” Langmuir 33, no. 8 (2016): 1891–1907, 10.1021/acs.langmuir.6b03606. 27983861

[49] M. D. Rao , K. K. Singh , C. A. Morrison , and J. B. Love , “Selective Recovery of Nickel from Obsolete Mobile Phone PCBs,” Hydrometallurgy 210 (2022): 105843, 10.1016/j.hydromet.2022.105843.

[50] W. Zhan , Y. Yuan , X. Yao , et al., “Efficient Recovery of Gold(I) From Thiosulfate Solutions Through Photocatalytic Reduction With Mn(II)‐Doped MoS_2_ ,” ACS Sustainable Chemistry & Engineering 9, no. 35 (2021): 11681–11690, 10.1021/acssuschemeng.1c02160.

[51] K. R. Kim , S. Choi , C. T. Yavuz , and Y. S. Nam , “Direct Z‐Scheme Tannin–TiO_2_ Heterostructure for Photocatalytic Gold Ion Recovery From Electronic Waste,” ACS Sustainable Chemistry & Engineering 8, no. 19 (2020): 7359–7370, 10.1021/acssuschemeng.0c00860.

[52] K. Babić , V. Tomašić , V. Gilja , et al., “Photocatalytic Degradation of Imidacloprid in the Flat‐Plate Photoreactor Under UVA and Simulated Solar Irradiance Conditions—The Influence of Operating Conditions, Kinetics and Degradation Pathway,” Journal of Environmental Chemical Engineering 9, no. 4 (2021): 105611, 10.1016/j.jece.2021.105611.

[53] L. Ling , H. Tugaoen , J. Brame , et al., “Coupling Light Emitting Diodes With Photocatalyst‐Coated Optical Fibers Improves Quantum Yield of Pollutant Oxidation,” Environmental Science & Technology 51, no. 22 (2017): 13319–13326, 10.1021/acs.est.7b03454. 29028332

[54] F. Zhao , D. Cambié , J. Janse , et al., “Scale‐Up of a Luminescent Solar Concentrator‐Based Photomicroreactor via Numbering‐up,” ACS Sustainable Chemistry & Engineering 6, no. 1 (2017): 422–429, 10.1021/acssuschemeng.7b02687. 29333350 PMC5762165

[55] M. Mann , T. P. Nicholls , H. D. Patel , et al., “Sustainable Gold Extraction from Ore and Electronic Waste,” Nature Sustainability 8, no. 8 (2025): 947–956, 10.1038/s41893-025-01586-w.

[56] K. Fu , X. Liu , X. Zhang , et al., “Utilizing Cost‐Effective Pyrocarbon for Highly Efficient Gold Retrieval From e‐Waste Leachate,” Nature Communications 15, no. 1 (2024): 6137, 10.1038/s41467-024-50595-4. PMC11271467 39033214

