# Supplementary material for: Autonomous Solar Metallurgy for High‐Purity Gold Recovery from Electronic Waste
Source: Adv Sci (Weinh). 2026 Sep 6:e77596. Online ahead of print. doi: 10.1002/advs.77596 (PMC13547716; doi:10.1002/advs.77596)
Supplement: Supplementary file 1 [file ADVS-9999-e77596-s002.docx]

Autonomous Solar Metallurgy for High-Purity Gold Recovery from Electronic Waste

Chaopeng Liu, Jiajun Li, Jialin Ruan, Wei Zhang, Wei Sun, Hongmei Li, Lei Xie,* and Min Liu*

Chaopeng Liu, Jiajun Li, Jialin Ruan, Wei Zhang, Prof. Wei Sun, Prof. Lei Xie

School of Minerals Processing and Bioengineering, Central South University

Changsha, 410083 (China)

E-mail: lei.xie@csu.edu.cn (L. Xie)

Hongmei Li, Prof. Min Liu

Hunan Joint International Research Center for Carbon Dioxide Resource Utilization, School of Physics, Central South University

Changsha, 410083 (China)

E-mail: minliu@csu.edu.cn (M. Liu)

Chaopeng Liu, Jiajun Li, Prof. Lei Xie

Furfure Resources Interface Science and Intelligent Application Research Center (FRISIARC)

Changsha, 410083 (China)

E-mail: lei.xie@csu.edu.cn (L. Xie)

Chaopeng Liu

Henan Key Laboratory of Water Pollution Control and Rehabilitation Technology, Henan University of Urban Construction

Pingdingshan, 467036 (China)

**Supplementary Methods 1**

**Materials**

Tetrachloroauric acid (HAuCl_4_, 97%) and disodium sulfide (Na_2_S) were purchased from Shanghai Haohong Biomedical Technology Co., Ltd. Sodium hydroxide (NaOH), ethylenediamine, N-bromosuccinimide, NaBr, and ascorbic acid were obtained from Sinopharm Chemical Reagent Co., Ltd. CuCl_2_·2H_2_O, pyridine, Na_2_S_2_O_3_ 5H_2_O, and polyvinylpyrrolidone (PVP) were supplied by Macklin Inc. trichloroisocyanuric acid, thiourea, HCl, and HNO_3_ were supplied by Shanghai Aladdin Biochemical Technology Co., Ltd. Sodium borohydride (NaBH_4_) was acquired from Shanghai Yien Chemical Technology Co., Ltd. All chemicals were of analytical grade and used without further purification.

**Synthesis of Cu_2_O**

Cu_2_O octahedrons were synthesized using a previously reported method with minor modifications. Specifically, 5 mL of NaOH solution (2 mol L^-1^) was slowly added dropwise to 50 mL of CuCl_2_·2H_2_O solution (0.01 mol L^-1^) containing 1.7 g of PVP, and the mixture was stirred for 30 min. Subsequently, 5 mL of ascorbic acid solution (0.6 mol L^-1^) was added to the mixed solution, and stirring continued for 180 min. The resulting suspensions were subjected to centrifugation at 8,000 r min^-1^ for 5 min to collect the products, which were then washed three times with deionized water and ethanol. Finally, Cu_2_O octahedrons were obtained after drying in a vacuum oven at 50 °C for 12 h.

**Synthesis of Cu_31_S_16_**

Cu_31_S_16_ was synthesized via a two-step method using Cu_2_O as a precursor. Initially, 60 mg of octahedral Cu_2_O was immersed in 10 mL of NaBH_4_ solution (0.5 mol L^-1^) for 4 h, followed by washing three times with ethanol to remove residual impurities and drying in a vacuum oven at 60 °C for 12 h. Then, the resulting product was dispersed in 60 mL of ultrapure water and sonicated for 30 min to form a black suspension. Under continuous stirring, 80 mL of Na_2_S solution (20 mmol L^-1^) was added to the suspension. After stirring for 30 min, the product was collected by centrifugation at 8000 rpm, thoroughly washed several times with ethanol and ultrapure water, and finally dried in a vacuum oven at 60 °C for 12 h.

**Synthesis of Cu_2_S**

Cu_2_S was synthesized via a modified hydrothermal method.^[^^1^^]^ Briefly, CuCl_2_ 2H_2_O (1 mmol) was dissolved in ethylenediamine (EDA, 30 mL), followed by the addition of thiourea (3 mmol) under continuous stirring. After 2 h of homogenization, the precursor solution was transferred into a 50 mL Teflon-lined autoclave and heated at 80 °C for 8 h. The resulting precipitates were collected after natural cooling, sequentially washed with deionized water and ethanol, and dried at 60 °C for 10 h prior to characterization.

**Characterization Methods**

The crystal structures of samples were identified by X-ray diffraction (XRD, D8 Advance, Bruker Corporation) with Co Kα radiation. The surface morphologies of Cu_16_S_31_ before adsorption and after adsorption were studied by scanning electron microscopy (SEM, signa 300 field emission, Zeiss). A BET analyzer (Micromeritics Gemini VII instrument) was used to determine the specific surface area of Cu_2_O and Cu_16_S_31_ from N_2_ adsorption-desorption isotherms at 77 K. The light absorption in the range of 200-2500 nm was recorded by ultraviolet-visible spectroscopy (Shimadzu, UV-3600) with an attached integrating sphere (ISR3100). UTi320E infrared camera was used to take infrared photographs. A power meter (CEL-NP2000-2A) was used to measure the light intensity. The Zeta potential of Cu_16_S_31_ were investigated via a Zetasizer Nano analyzer (ZS90, Malvern Instruments Inc.) over a pH range of 2.0-10.0. The elemental composition and chemical state changes of Cu_16_S_31_ before and after adsorption, along with the valence state of the adsorbed gold, were further analyzed by X-ray photoelectron spectroscopy (XPS, Escalab X, Thermo Fisher) to gain deeper insights into the adsorption-reduction mechanism. The concentration of remaining Au(III) and other metal elements was determined by inductively coupled plasma optical emission spectrometry (ICP-OES, PerkinElmer Avio 500).

**Supplementary Methods 2**

**Gold recovery performance investigation**

In a typical batch adsorption experiment, 20 mg of Cu_16_S_31_ was added to 50 mL of an aqueous Au(III) solution at a specified initial concentration in the dark and under 1 kW m^-2^ simulated sunlight illumination. The temperatures of Au(III) solution under light and under dark were controlled at the same 25 °C. After reaching adsorption equilibrium, the solids were separated by filtration through a 0.22 μm membrane. To ensure the reliability of the experimental results, all experiments in this study were conducted with three replicate samples. The adsorption capacity (Q, mg g^-1^) and adsorption rate (R, %) of adsorbent were obtained by the following Eqs. (1)-(2):

$$\text{Q}\text{=}\frac{\text{(C}_{\text{0}}\text{-}\text{C}_{\text{e}}\text{)V}}{\text{M}}\text{ (1)}$$

$$\text{R=}\frac{\text{C}_{\text{0}}\text{-}\text{C}_{\text{e}}}{\text{C}_{\text{0}}}\text{×100\% (2)}$$

Where C_0_ (mg·L^-1^) and C_e_ (mg·L^-1^) are the initial and equilibrium concentrations of Au(III) in solution. V (L) is the volume of Au(III) solution. M (g) is the mass of adsorbent.

**2.1 Adsorption isotherms**

Adsorption isotherms were constructed using initial Au(III) ions concentrations ranging from 50 to 3500 mg L^-1^ to evaluate the saturation adsorption capacity of Cu_16_S_31_. To verify the adsorption mechanism of Au(III) on Cu_16_S_31_, the Langmuir (Eq. (3)) and Freundlich models (Eq. (4)) were employed to fitting the experimental data, respectively. The linear equations of each model were as follows^[^^2^^]^:

$$\frac{\text{C}_{\text{e}}}{\text{Q}_{\text{e}}}\text{=}\frac{\text{C}_{\text{e}}}{\text{Q}_{\text{max}}}\text{+}\frac{\text{1}}{\text{Q}_{\text{max}}\text{K}_{\text{L}}}\text{ (3)}$$

$$\text{Q}_{\text{e}}\text{=}\text{K}_{\text{F}}{\text{C}_{\text{e}}}^{\frac{\text{1}}{\text{n}}}\text{ (4)}$$

Where C_e_ (mg L^-1^) and Q_e_ (mg g^-1^) are the concentration of Au(III) and the amount of Au(III) adsorbed by adsorbent at equilibrium time respectively, Q_max_ (mg g^-1^) is the maximal amount of Au(III) adsorbed by adsorbent. K_L_ (L mg^-1^) and K_F_ (L mg^-1^) are the Langmuir equilibrium constant and Freundlich equilibrium constant, respectively.

**2.2 Adsorption kinetics**

Adsorption kinetics were evaluated at an initial Au(III) ions concentration of 100 mg L^-1^. For each experiment, 20 mg of Cu_31_S_16_ was added to 50 mL of the Au(III) solution. The suspension was continuously stirred at 300 rpm throughout the experiment. For the light-on experiment, the incident light intensity was measured using a power meter (CEL-NP2000-2A) and stabilized at 1 kW m^-2^ for 5 min before each experiment. The reaction was initiated by adding Cu_31_S_16_ to the Au(III) solution This moment was defined as t=0. For the light-off experiment, the adsorbent dosage, solution volume, reactor geometry, stirring rate, and sampling procedure were identical to those used in the light-on experiment, except that the reactor was shielded from illumination using aluminum foil. At predetermined time intervals, 2 mL of the solution was withdrawn using a 5 mL syringe and immediately filtered through a 25 mm hydrophilic PTFE syringe filter with 0.22 μm pores. The filtrate was subsequently analyzed for residual Au concentration by ICP-OES. All experiments were performed in triplicate to ensure reliability. The pseudo-first-order (PFO, Eq. (5)) and the pseudo-second-order (PSO, Eq. (6)) were employed to fitting the experimental data, respectively. Their curvilinear equations obeyed (5) and (6)^[^^3^^]^:

$$\text{ln(Q}\text{e}\text{-Q}\text{t}\text{)=lnQ}\text{e}\text{-K}\text{1}\text{t }\text{ (5)}$$

$$\frac{\text{t}}{\text{Q}_{\text{t}}}\text{=}\frac{\text{1}}{\text{K}_{\text{2}}{\text{Q}_{\text{e}}}^{\text{2}}}\text{+}\frac{\text{t}}{\text{Q}_{\text{e}}}\text{ }\text{ (6)}$$

Where K_1_ (min^-1^) and K_2_ (g mg·min^-1^) are the PFO and PSO constant, respectively. Q_t_ (mg·g^-1^) is the adsorption capacity at time t. The Cu-release/Au-recovery mass ratio during Au(III) adsorption was calculated using Eqs. (7)-(9):

*m*_Cu, release_ = *C*_Cu, release_ * *V*	(7)

*m*_Au, recovered_ = (*C*_Au, initial_ - *C*_Au, final_)* *V*	(8)

*R*_Cu, release/Au, recovered_ =$\frac{\text{m}_{\text{Cu, release}}}{\text{m}_{\text{Au, recovered}}}\text{×100\%}$ (9)

**2.3 Adsorption thermodynamics under dark**

The Van't Hoff equation was used to determine whether the adsorption process in the absence of light was an endothermic reaction. The enthalpy change (∆H), entropy change (∆S), and Gibbs free energy change (∆G) were calculated using Eqs. (10) and (11):

$$\ln\text{K=-}\frac{\text{∆}\text{H}}{\text{RT}}\text{+}\frac{\text{∆}\text{S}}{\text{R}}\text{ (10)}$$

∆G=∆H-T∆S	(11)

Where K was the distribution coefficient, calculated from the ratio of the adsorption capacity of the equilibrium reaction to the concentration of the equilibrium reaction, T was the kelvin temperature (k), and R was the gas constant (8.314 J mol^-1^ k^-1^).

**2.4 Adsorption selectivity**

Binary and multicomponent metal ion solutions were employed to evaluate the adsorption selectivity of Cu_31_S_16_ toward Au(III). Competing ions, including Na(I), K(I), Ca(II), Mg(II), Cd(II), Pb(II), Cr(VI), Mn(II), Fe(III), Ni(II), Co(II), Zn(II), Cu(II), Pd(II), and Ag(I), were introduced as their nitrate salts. In binary-metal ion mixed solution, the initial concentrations of competitive ions and Au(III) was 100 and 10 mg L^-1^, respectively. A 20 mg of Cu_16_S_31_ was added to 50 mL of mixed solution without pH adjustment under 1 kW m^-2^ simulated sunlight illumination. The principal adsorbed ions can be determined by the distribution coefficient (K_d_) and separation factor (SF),^[^^4^^]^ calculated using Eqs. (12) and (13):

$$\text{K}_{\text{d}}\text{=}\frac{\text{C}_{\text{0}}\text{-}\text{C}_{\text{e}}}{\text{C}_{\text{e}}}\text{*}\frac{\text{V}}{\text{M}}\text{ (12)}$$

$$\text{SF}\text{=}\frac{\text{K}_{\text{d}}\text{(Au(III))}}{\text{K}_{\text{d}}\text{(other metal ions)}}\text{ (13)}$$

Where C_0_ (mg·L^-1^) and C_e_ (mg·L^-1^) are the initial and equilibrium concentrations of Au(III) in solution. V (L) was the volume of Au(III) solution. M (g) was the mass of adsorbent.

**2.5 Optimal pH range and reusability**

To determine the optimal pH for gold uptake, the pH of the Au(III) solution was adjusted to 1.0-10.0 using 0.1 mol L^-1^ HCl or NaOH solutions. Subsequently, 20 mg of Cu_16_S_31_ adsorbent was added to 50 mL of a 100 mg L^-1^ Au(III) solution, and gold uptake experiments were conducted at 25 °C under 1 kW m^-2^ simulated sunlight illumination. The repeatability of Cu_16_S_31_ was assessed using 1 M thiourea solution (pH = 2.0) as the eluent.

**Supplementary Methods 3**

**Gold uptake via breakthrough experiments**

Continuous-flow adsorption experiments were conducted using a vertical glass column with an inner diameter of 10 mm and a length of 1 cm. Cu_31_S_16_ (0.20 g) was uniformly loaded into the column, and a 0.45 μm membrane was placed at the bottom to retain the adsorbent and prevent particle loss. Before each experiment, the adsorption column was rinsed with excess deionized water. The column was irradiated using an AM 1.5G solar simulator at an incident light intensity of 1.0 kW m^-2^, as measured at the external surface of the column using a calibrated power meter. The conductive pins were manually removed from discarded AMD CPUs using pliers, washed thoroughly with deionized water, and dried. The cleaned conductive pins were subsequently leached with aqua regia, and the resulting leachate was diluted tenfold with deionized water. A mixed influent solution containing 173.1 mg L^-1^ Au(III), 1589.6 mg L^-1^ Cu(II), and 789.4 mg L^-1^ Ni(II) was continuously introduced into the adsorption column in the bottom-to-top direction using a peristaltic pump at a flow rate of 2.0 mL min^-1^. Effluent fractions of 2 mL were collected at predetermined time intervals. Each fraction was immediately filtered through a 0.22 μm hydrophilic PTFE membrane and appropriately diluted before the concentrations of Au, Co, and Ni were determined by ICP-OES. The breakthrough behavior was evaluated by plotting (C_t_/C_0_) as a function of operation time or cumulative effluent volume, where (C_0_) and (C_t_) represent the influent and effluent concentrations, respectively. The cumulative dynamic Au uptake was calculated according to^[^^5^^]^:

$\text{q}=\frac{\text{C}_{\text{0}}\text{F}}{\text{1000m}}\int_{\text{0}}^{\text{t}} \text{(1-}\frac{\text{C}_{\text{t}}}{\text{C0}}\text{) dt}$	(14)

where q represents the amount of metal retained (mg g^-1^), F is the flow rate (mL min^-1^), m is the mass of sorbent in the column (g) and t is the sorption time (min).

**Supplementary Methods 4**

**Gold recovery from electronic waste**

Electronic wastes were obtained from a waste computer AMD CPU (4 cm × 4 cm). The conductive pins were manually removed from the AMD CPU using pliers. First, coupled scanning electron microscopy and energy-dispersive X-ray spectroscopy (SEM-EDS) established Cu, Ni, and Au as the predominant metallic species in the conductive pins. Subsequently, the Au and other metals were leached using aqua regia, which was prepared by mixing concentrated HCl and HNO_3_ in a 3:1 volume ratio. The leaching solution gradually changed from yellow to dark green and was then subjected to elemental analysis by ICP-OES. Afterward, 10 mg of Cu_31_S_16_ was added to 25 mL of the leaching solution until adsorption equilibrated. 2 mL solution were withdrawn using a 5 mL syringe and immediately filtered through a 25 mm hydrophilic PTFE syringe filter with 0.22 μm pores to ascertain the concentration of Au(III) and other competing metal ions. Additionally, other authentic e-waste streams, including mobile phone motherboards, were evaluated using the same adsorption protocol employed for the AMD CPU-derived leachate.

The Au-recovery performance of Cu_31_S_16_ was further evaluated using different leach solutions, including thiosulfate, trichloroisocyanuric acid (TCCA), and N-bromosuccinimide/pyridine (NBS/Py) systems. For thiosulfate leaching, conductive pins (0.10 g) were immersed in 20 mL of a lixiviant containing 0.2 M Na_2_S_2_O_3_ 5H_2_O and 10 mM ethylenediamine (en), with the pH adjusted to 11. The mixture was shaken at 200 rpm and 25 °C for 48 h. After leaching, the suspension was filtered through a 0.22 μm membrane, and the concentrations of dissolved Au and other metals in the resulting pregnant leach solution were determined by ICP-OES. For TCCA leaching, conductive pins (0.10 g) were added to 10 mL of deionized water containing TCCA (0.593 g) and NaBr (8.7 mg).^[^^6^^]^ The mixture was stirred at 200 rpm and 25 °C in a vented plastic vessel. After 2 h, an additional 17.4 mg of NaBr was added, and leaching was continued for a total of 5 h. The resulting suspension was filtered through a 0.22 μm membrane, and the concentrations of dissolved Au and other metals in the pregnant leach solution were determined by ICP-OES. For a milder leaching approach, an aqueous NBS/Py system was employed. Specifically, 0.960 mL of pyridine (100 mM) and 0.750 g of NBS (35 mM) were mixed with 119 mL of distilled water to prepare the leaching solution.^[^^7^^]^ Subsequently, 0.1 g of CPU conductive pins was added to the leaching solution and allowed to react for 24 h. The initially colorless solution gradually turned blue during leaching. After the reaction, the leachate was filtered through a 0.22 μm membrane, and the concentrations of Au and coexisting metal ions were determined by ICP-OES. Afterward, 10 mg of Cu_31_S_16_ was added to 25 mL of the leaching solution and allowed to adsorb until equilibrium was reached. 2 mL solution were withdrawn using a 5 mL syringe and immediately filtered through a 25 mm hydrophilic PTFE syringe filter (0.22 μm pores size) to determine the concentration of remaining Au(III) and other competing metal ions by ICP-OES.

**Supplementary Methods 5**

**Gold separation and purification**

Gold was separated and purified from the Au-loaded Cu_31_S_16_ using a combined acid-washing and calcination procedure.^[^^8^^]^ Au-loaded Cu_31_S_16_ (100 mg) was dispersed in 10 mL of concentrated HCl (37 wt%) and stirred at 200 rpm and room temperature for 24 h to remove Cu-containing and other acid-soluble impurities. The solid was collected by filtration, and the HCl treatment was repeated once using a fresh 10 mL aliquot of concentrated HCl. After acid treatment, the recovered solid was washed three times with deionized water and then dried at 60 °C. The dried solid was placed in an alumina boat and heated in a tube furnace under an air atmosphere from room temperature to 900 °C at a heating rate of 10 °C min^-1^. The temperature was maintained at 900 °C for 2 h, after which the furnace was allowed to cool naturally to room temperature, yielding metallic gold powder. For purity analysis, a known mass of the recovered gold powder was completely dissolved in freshly prepared aqua regia and diluted to a defined volume. The concentrations of residual impurity elements were determined by ICP-OES, and the gold purity was tested referring to the standard GB/T 38145-2019.

**Supplementary Methods 6**

**Details of technoeconomic analysis (TEA)**

A preliminary process-level techno-economic analysis was performed following the cost-accounting framework reported by Fu et al.^[^^8^^]^ The analysis considered three principal process modules: leaching of the CPU feedstock, production of Cu_31_S_16_, and gold capture, separation, and purification. By referring to the Guidelines for Techno-Economic Analysis of Adsorption Processes written by Danaci et al,^[^^9^^]^ the production step of Cu_31_S_16_ could meet the industrial suitability, taking into account the required characteristics (e.g., water tolerance, rapid mass transfer, low cost, low environmental and safety impact, etc.). Material ratios, operating conditions, processing time, electricity consumption, and product yields were derived from the laboratory-scale experiments unless otherwise specified. The methods for the leaching of CPU scrap and the purification of recovered gold were referenced from previous literature.^[^^10^^]^ Of note, both the electricity price range adopted in this work (0.02 ~ 0.06 $ kWh^-1^) and the exclusion of upstream collection, dismantling, crushing, and plastic-metal separation from the system boundary followed the TEA framework previously reported for pyrocarbon-based gold recovery.^[^^8^^]^ Notably, electricity costs in our work were found to be lower than material and labor costs, in agreement with earlier reports.^[^^11^^]^ Labor costs were estimated according to the 2022 global average monthly wage, with reference to both low- and high-income countries. It was found that labor costs have a greater impact on total cost when a larger workforce is employed, implying that the rational siting of the production facility is essential for cost control and profitability. However, as CPU scrap constitutes a major cost driver in gold recovery, its market price exhibits considerable volatility. This fluctuation stems from variations in scrap grade and gold content, as well as the diverse procurement channels ranging from retailers and hawkers to formal recycling organizations. On the basis of both market assessments and reported literature values, a price of US$ 13.72 per kg has been adopted for CPU scrap, exceeding typical wholesale purchase rates.^[^^12-13^^]^

Aqua regia was used as the laboratory-scale benchmark lixiviant. In the baseline calculation, HCl and HNO_3_ were treated as once-through reagents, and no recovery or reuse of their acid components was assumed. The direct costs of HCl, HNO_3_, and process water were included. Industrial-scale aqua-regia leaching would additionally require corrosion-resistant reactors and piping, off-gas capture and scrubbing, neutralization, wastewater treatment, and residual-metal management. Because pilot-scale material and equipment inventories for these auxiliary operations were unavailable, these costs were not monetized and the reported value should therefore be regarded as a lower-bound process-cost estimate. To specify the economic potential of the gold recovery process using Cu_31_S_16_ we proposed, 1 ton of CPU scraps being recovered was took as an example. In accordance with the actual experimental conditions during our lab-scale production of Cu_31_S_16_, the calculation details such as material ratios and reaction parameters were carefully selected. No regeneration or reuse credit was assumed, corresponding to complete replacement of the material after each functional unit. A comprehensive analysis of the material flow and associated costs throughout the gold recovery process was conducted to offer valuable insights for future research. However, the actual profitability remains to be validated through large-scale trials. Specific assumptions underlying the cost calculations will be discussed in detail below.

(1) Capital cost. The total capital cost comprises the purchased equipment costs and other indirect capital expenses. The other capital costs include equipment installation, process piping, instrumentation and control systems, and electrical systems, etc. Because these costs depend strongly on process configuration, plant scale, and site-specific conditions, they are difficult to determine directly and are commonly estimated using percentage factors based on the purchased equipment cost. Accordingly, the capital cost was estimated following the methodology previously reported for a pyrolytic-carbon-based gold recovery process.^[^^8^^]^

1. Equipment purchase cost:

= $27.43 (laboratory water bath) + $518.83 (centrifuge) + $20.58 (Magnetic stirrer) + $607.64 (Teflon-lined autoclave) + $846.09 (tube furnace) + $548.70 (thermostatic oscillator) + $41.2 (glass wares) = $2610.47

2. Other capital costs: The other capital costs were calculated by assuming them 57.84% of the total fixed capital estimate as reported by Yunus and coworkers.^[^^14^^]^ Therefore, the other capital costs can be calculated to be $3581.35.

(2) Operating costs. The operating costs comprised electricity, maintenance, labor, depreciation, and other operating costs (e.g., administrative cost, insurance fee, etc.). The electricity price was set at US$0.06 kWh^-1^ based on the combined peak- and off-peak industrial electricity price in Changsha, China. Annual maintenance costs were assumed to be 2.0% of the purchased equipment cost.^[^^15^^]^ Labor costs were estimated using the average wage in China in 2022 together with the required staffing level. Depreciation, representing the annual allocation of fixed capital over the service life of the plant, was calculated using a straight-line rate of 10% of the fixed capital per year, following a previous techno-economic analysis of rice-husk-derived adsorbent production.^[^^16^^]^ Administrative expenses, including costs associated with administrative personnel, office operation, and sales support, were estimated as 45.0% of the labor cost.^[^^8^^]^ Insurance costs associated with the plant and products were assumed to be 1.0% of the fixed capital.^[^^8^^]^

1. Electricity:

= 0.06 $ kWh^-1^ × 356.167 kWh = $21.37

2. Maintenance:

= $2610.47 (equipment purchase cost) × 2.0% = $52.21

3. Labor:

= 876.02 $ month^-1^ × 12 month = $10512.24

4. Depreciation:

= $6191.82 × 10.0% = $619.18

5. Other operating cost:

= 876.02 $ month^-1^ × 12 month × 45.0% (administrative cost) + $6191.82 × 1.0% (Insurance fee) = $4792.43

(3) Material costs. The prices of all the raw chemicals and reagents were based on industrial grade purity reagents, and were obtained from the recent prices on the official website of Baidu Alibaba International Shopping (https://b2b.baidu.com), the local suppliers, and also the previous studies.^[^^8^^]^ The annual usage of these chemicals were calculated based on their daily consumption in the lab-scale production processes.

1. Cu_2_O:

= 23.608 $ kg^-1^ × 0.383 kg = $9.04

2. NaBH_4_:

= 259.463 $ kg^-1^ × 0.0202 kg = $5.24

3. Na_2_S:

= 3594.015 $ kg^-1^ × 0.102 kg = $366.59

4. CPU scrap:

= 13.717 $ kg^-1^ × 1000 kg = $13717.00

5. Other chemicals (Water, HCl, HNO_3_):

= (0.0850 $ kg^-1^ × 186.800 kg) + (0.0220 $ kg^-1^ × 24.22 kg) + (0.210 $ kg^-1^ × 21.79 kg) = $20.99

(4) Total cost

= ($2610.47 + $3581.35) + ($21.37 + $52.21 + $10512.24 + $619.18 + $4792.43) + ($9.04 + $5.24 + $366.59 + $13717.00 + $20.99) = $36308.11

**Supplementary Methods 7**

**Density functional theory (DFT) calculation parameter settings**

All DFT calculations were carried out with the CP2K 2024.1 package and its Quickstep module, which utilizes a combined Gaussian and plane-wave (GPW) approach. Geometry optimizations were performed in a fully periodic three-dimensional setting via the BFGS algorithm, limited to a maximum of 2000 steps. The exchange-correlation energy was described using the Perdew-Burke-Ernzerhof (PBE) functional under the generalized gradient approximation (GGA), augmented with Grimme’s DFT-D3 dispersion correction for long-range van der Waals interactions. Atomic cores were represented by Goedecker-Teter-Hutter (GTH) pseudopotentials, paired with DZVP-MOLOPT-SR-GTH basis sets. A plane-wave cutoff energy of 400 Ry was applied for the electron density. The self-consistent field procedure employed an orbital transformation (OT) method with a full single‑step inverse preconditioner and conjugate gradient minimizer. The SCF convergence threshold was set to 1 × 10^-5^ Hartree, with up to 300 inner and 100 outer SCF cycles. An initial guess was generated by restarting from a prior wavefunction, and electron density extrapolation was conducted using the ASPC scheme with a second-order polynomial.

The simulation cells were constructed based on the optimized lattice parameters of the Cu_31_S_16_ (001) surface, with the in-plane lattice vectors defined as A = (12.051, 0.000, -6.006) Å and B = (0.000, 15.745, 0.000) Å, and the surface-normal vector C = (34.047, 0.000, 68.318) Å. A vacuum layer of 30 Å was introduced along the surface normal (z direction) to eliminate spurious interactions between periodically repeated slabs. All surface atoms and AuCl_x_ adsorbates were fully relaxed until the geometry optimization convergence criteria were satisfied. For single-point energy calculations, the basis set for non-metal atoms was upgraded from DZVP-MOLOPT-SR-GTH to TZV2PX-MOLOPT-GTH to improve the accuracy of adsorption energy evaluations, while metal atoms were described using the DZVP-MOLOPT-SR-GTH basis set; all other computational parameters, including the exchange-correlation functional, dispersion correction, pseudopotentials, SCF settings, and convergence thresholds, were kept identical to those employed in the geometry optimization stage.

**Supplementary Figure 1.** SEM image of Cu_2_O octahedron particles.

**Supplementary Figure 2.** SEM image and EDS mapping of Cu_31_S_16_.

**Supplementary Figure 3.** N_2_ adsorption and desorption isotherms at 77 K for Cu_2_O and Cu_31_S_16_.

**Supplementary Figure 4.** Pore size distributions of Cu_2_O and Cu_31_S_16_.

**Supplementary Figure 5.** The appearance color of Cu_2_O and Cu_31_S_16_.

**Supplementary Figure 6.** The Tauc plot curves of Cu_2_O (blue) and Cu_31_S_16_ (red).

**Supplementary Figure 7.** Infrared photographs of Cu_2_O surface against time under 1-sun irradiation.

**Supplementary Figure 8.** Surface temperature of Cu_2_O and Cu_31_S_16_ as a function of time under 1-sun irradiation.

**Supplementary Figure 9.** Mott-Schottky plots of Cu_31_S_16_.

**Supplementary Figure 10.** Nyquist plots of Cu_31_S_16_ under dark and under 1-sun irradiation.

**Supplementary Figure 11.** Photocurrent response curves of Cu_2_O and Cu_31_S_16_.

**Supplementary Figure 12.** Van't Hoff plots for Au(III) adsorption on Cu_31_S_16_ (initial concentrations of Au(III) was 1200-2000 mg L^–1^; adsorbent dose 0.4 g L^–1^; solution volume 50 mL; without pH adjustment and the temperature was 25, 35 and 45 °C).

**Supplementary Figure 13.** Au(III) adsorption efficiency of Cu_31_S_16_ at 50 mg L^-1^ under dark and illuminated conditions (bulk temperature: 25 °C).

**Supplementary Figure 14.** Au(III) uptake by Cu_31_S_16_ at varying concentrations under dark and illuminated conditions (bulk temperature: 25 °C).

**Supplementary Figure 15.** Cu-release/Au-recovery mass ratio and Au(III) adsorption from an initial concentration of 100 mg L^-1^.

**Supplementary Figure 16.** Cu-release/Au-recovery mass ratio and Au(III) adsorption from an initial concentration of 500 mg L^-1^.

**Supplementary Figure 17.** XRD patterns of Cu_2_S.

**Supplementary Figure 18.** Comparative adsorption kinetics of Cu_2_S and Cu_31_S_16_ toward Au(III) at an initial concentration of 100 mg L^-1^.

**Supplementary Figure 19.** Comparative adsorption capacities of Cu_2_S and Cu_31_S_16_ toward Au(III).

**Supplementary Figure 20.** Effect of competitive ions on Au(III) adsorption in binary-metal ion mixed solution (initial concentrations of competitive ions and Au(III) was 100 and 10 mg L^-1^, respectively; adsorbent dose 0.4 g L^–1^; solution volume 50 mL; without pH adjustment and 1-sun irradiation).

**Supplementary Figure 21.** XRD patterns of pristine and regenerated Cu_31_S_16_.

**Supplementary Figure 22.** Au(III) removal efficiency of Cu_31_S_16_ over ten sequential adsorption runs at an initial Au concentration of 100 mg L^-1^.

**Supplementary Figure 23.** Au removal efficiency at pH 1 from a 50 mg L^-1^ Au(III) solution.

**Supplementary Figure 24.** Cu dissolution from Cu_31_S_16_ as a function of pH (pH 1-6).

**Supplementary Figure 25.** XRD patterns of pristine Cu_31_S_16_ and Cu_31_S_16_ after exposure to pH 1 solution.

**Supplementary Figure 26.** SEM image and EDS elemental mapping of Cu_31_S_16_ after exposure to pH 1 solution.

**Supplementary Figure 27.** High-resolution Au 4f XPS spectra of Cu_31_S_16_-Au-D and Cu_31_S_16_-Au-L after adsorption tests. The deconvolution identifies the characteristic peaks of Au(0) (red) and Au(I) (blue).

**Supplementary Figure 28.** High-resolution Cu 2p XPS spectra of Cu_31_S_16_-Au-D and Cu_31_S_16_-Au-L after adsorption tests. The deconvolution identifies the characteristic peaks of Cu(II) (blue) and Cu(I) (red).

**Supplementary Figure 29.** High-resolution S 2p XPS spectra of Cu_31_S_16_-Au-D and Cu_31_S_16_-Au-L after adsorption tests.

**Supplementary Figure 30.** Concentrations of metal ions in aqua regia leachates from AMD CPU pins before and after adsorption using Cu_31_S_16_.

**Supplementary Figure 31.** Concentrations of metal ions in trichloroisocyanuric acid (TCCA) leachates from AMD CPU pins before and after adsorption using Cu_31_S_16_.

**Supplementary Figure 32.** Concentrations of metal ions in N-bromosuccinimide/pyridine (NBS/Py) leachates from AMD CPU pins before and after adsorption using Cu_31_S_16_.

**Supplementary Figure 33.** Concentrations of metal ions in thiosulfate leachates from AMD CPU pins before and after adsorption using Cu_31_S_16_.

**Supplementary Figure 34.** Concentrations of metal ions in aqua regia leachates derived from mobile phone motherboards before and after adsorption using Cu_31_S_16_.

**Supplementary Table 1.** Thermodynamic parameters for Au(III) adsorption on Cu_31_S_16_.

| ∆Hº  (kJ mol^-1^) | ∆Sº  (kJ mol^-1^ K^-1^) | R^2^ | ∆Gº (kJ mol^-1^) | | |
| --- | --- | --- | --- | --- | --- |
|  |  |  | 25 °C | 35 °C | 45 °C |
| 4.79 | 0.033 | 0.999 | -5.00 | -5.32 | -5.67 |

**Supplementary Table 2.** Kinetic parameters for 100 mg L^-1^ Au(III) adsorption on Cu_31_S_16_ under dark and under light irradiation.

|  | pseudo-first-order | | | pseudo-second-order | | |
| --- | --- | --- | --- | --- | --- | --- |
|  | K_1_ | Q_e,cal_ | R^2^ | K_2_ | Q_e,cal_ | R^2^ |
| Dark | 0.023 | 168.27 | 0.920 | 1.248×10^-4^ | 203.84 | 0.958 |
| 1-sun irradiation | 0.042 | 244.965 | 0.965 | 1.923×10^-4^ | 278.02 | 0.974 |

**Abbreviations:** Q_e,cal_, calculated adsorption capacity; K_1_, pseudo-first-order kinetic rate constant; K_2_, pseudo-second-order rate constant.

**Supplementary Table 3.** Isotherm model parameters for Au(III) adsorption on Cu_31_S_16_ under light irradiation.

| Models | Parameter | |
| --- | --- | --- |
| Langmuir | Q_m_ (mg·g^-1^) | 6146.44 |
|  | K_L_ (L·g^-1^) | 0.517 |
|  | R^2^ | 0.887 |
| Freundlich | K_F_ | 4035.32 |
|  | 1/n | 0.065 |
|  | R^2^ | 0.993 |

**Abbreviations:** Q_m_, calculated maximal adsorption capacity; K_L_, Langmuir equilibrium constant; K_F_, Freundlich equilibrium constant.

**Supplementary Table 4.** Effect of competitive ions on Au(III) adsorption in binary-metal ions mixed solution (initial concentrations of competitive ions and Au(III) was 100 and 10 mg L^-1^; adsorbent dose 0.4 g L^–1^; solution volume 50 mL; without pH adjustment and 1-sun irradiation).

| Interfering ions | C_0_  (mg L^-1^) | C_e_  (mg L^-1^) | R (%) | Au(III) | | |
| --- | --- | --- | --- | --- | --- | --- |
|  |  |  |  | C_0_  (mg L^-1^) | C_e_  (mg L^-1^) | R (%) |
| Ca(II) | 102.87 | 102.49 | 0.37 | 10.10 | 0.01 | 99.90 |
| Mg(II) | 99.01 | 98.64 | 0.37 | 9.96 | 0.02 | 99.80 |
| Cu(II) | 101.78 | 98.06 | 3.65 | 9.98 | 0 | 100 |
| Pb(II) | 98.89 | 98.23 | 0.67 | 9.98 | 0.01 | 99.90 |
| Cr(III) | 100.99 | 100.62 | 0.37 | 9.99 | 0 | 100 |
| Mn(II) | 99.45 | 99.09 | 0.36 | 10.30 | 0.03 | 99.71 |
| Fe(III) | 101.2 | 100.5 | 0.69 | 9.97 | 0 | 100 |
| Ni(II) | 98.56 | 97.87 | 0.70 | 10.04 | 0.02 | 99.80 |
| Co(II) | 99.77 | 99.03 | 0.74 | 9.98 | 0.01 | 99.90 |
| Zn(II) | 101.55 | 100.98 | 0.56 | 10.03 | 0.02 | 99.80 |
| Cd(II) | 98.72 | 98.27 | 0.46 | 9.97 | 0.01 | 99.90 |
| Ag(I) | 99.40 | 43.57 | 56.17 | 10.06 | 0 | 100 |
| Pd(II) | 98.67 | 30.67 | 68.92 | 9.97 | 0 | 100 |

**Abbreviations:** C_0_, initial concentration; C_e_, equilibrium concentration；R, removal efficiency.

**Supplementary Table 5.** The distribution coefficient (K_d_) and separation factor (SF) values of Cu_31_S_16_ in the mixed solutions containing multi-metal ions at a competing ions-to-Au concentration ratio of 10:1 (adsorbent dose 0.4 g L^-1^; solution volume 50 mL; without pH adjustment and 1-sun irradiation).

| Metal ions | R (%) | K_d_ (mL g^-1^) | SF | C_0_ (mg L^-1^) | C_e_ (mg L^-1^) |
| --- | --- | --- | --- | --- | --- |
| Cu(II) | 4.10 | 110 | 2.3×10^5^ | 100.56 | 96.44 |
| Pb(II) | 0.90 | 23 | 1.1×10^6^ | 98.91 | 98.02 |
| Cr(III) | 0.24 | 5.9 | 4.2×10^6^ | 101.28 | 101.04 |
| Mn(II) | 0.22 | 5.5 | 4.5×10^6^ | 99.74 | 99.52 |
| Fe(III) | 0.37 | 9.2 | 2.7×10^6^ | 100.88 | 100.51 |
| Ni(II) | 0.52 | 13 | 1.9×10^6^ | 99.22 | 98.7 |
| Co(II) | 0.76 | 19 | 1.3×10^6^ | 98.63 | 97.88 |
| Zn(II) | 1.24 | 32 | 7.8×10^5^ | 98.84 | 97.61 |
| Cd(II) | 1.17 | 29 | 8.6×10^5^ | 99.55 | 98.39 |
| Ag(I) | 53.19 | 4.29×10^3^ | 5.8×10^3^ | 100.01 | 46.81 |
| Pd(II) | 70.31 | 2.09×10^4^ | 1.2×10^3^ | 99.98 | 29.68 |
| Au(III) | 99.99 | 2.5×10^7^ | — | 10.07 | 0.01 |

**Abbreviations:** C_0_, initial concentration; C_e_, equilibrium concentration；R, removal efficiency.

**Supplementary Table 6.** Maximum adsorption capacity (Q_max_) and distribution coefficient (K_d_) for the adsorption of Au(III) on various adsorbents.

| Number | Adsorbents | Q_max_  (mg g^-1^) | K_d_  (mL g^-1^) | Competing ions | Cycle  numbers | Ref. |
| --- | --- | --- | --- | --- | --- | --- |
| 1 | CMTU | 3180 | 4.5×10^5^ | Cu(II), Ni(II), Cd(II), Al(III), Pb(II), Mn(II) | 5 | ^[^^17^^]^ |
| 2 | Cu_2_O-TU | 1529 | 1.3×10^5^ | Cd(II), Mn(II), Cu(II), Pb(II), Ni(II) | 5 | ^[^^18^^]^ |
| 3 | MPVA | 1425 | 8.1×10^6^ | Pt(IV), Pd(II), Fe(III), Co(II), Ni(II), Cu(II), Zn(II), Cd(II), Pb(II) | 10 | ^[^^19^^]^ |
| 4 | Fe-BTC/pTA | 920 | 3.8×10^5^ | Na(I), K(I), Co(II), Ni(II), Cu(II), Zn(II), Ca(II) | 5 | ^[^^7^^]^ |
| 5 | JNM-103 | 2324 | 1.9×10^6^ | K(I), Na(I), Mg(II), Al(III), Zn(II), Cu(II), Cd(II), Mn(II), Ni(II), Cr(III), Co(II), Ca(II), Fe(III) | 5 | ^[^^20^^]^ |
| 6 | JNM-100-AO | 954 | 2.9×10^6^ | Cu(II), Cr(III), Cd(II), Zr(IV), Fe(II), Hg(II), Na(I), K(I), Mn(II), Mg(II), Ca(II), Fe(III), Zn(II), Pt(II), Al(III), Ni(II), Ba(II), Pb(II), Pd(II) | 5 | ^[^^21^^]^ |
| 7 | FeCo-MOF-74 | 3078 | 1.3×10^7^ | Cu(II), Ni(II), Co(II), Cd(II), Zn(II) | 6 | ^[^^22^^]^ |
| 8 | Ionic-COF-Cl | 1271 | 9.0×10^5^ | Co(II), Cu(II), Fe(III), K(I), Mg(II), Na(I), Al(III), Ni(II), Zn(II) | 10 | ^[^^23^^]^ |
| 9 | COF-HNU-25 | 1725 | 6.9×10^6^ | Pd(II), Zn(II), Al(III), Ni(II), Cu(II), Fe(III) | 20 | ^[^^24^^]^ |
| 10 | TpDa-COF | 985 | 1.3×10^4^ | Ag(I), Cs(I), Ba(II), Ca(II), Cd(II), Co(II), Cu(II), Fe(II), Hg(II), Mg(II), Mn(II), Ni(II), Pb(II), Sr(II), Zn(II), Al(III), Cr(III), Fe(III) | 6 | ^[^^25^^]^ |
| 11 | IPcomp-9 | 2349 | 1.2×10^5^ | Na(I), K(I), Mg(II), Ca(II), Cr(III), Mn(II), Co(II), Ni(II), Cu(II), Fe(III), Pb(II) | 5 | ^[^^26^^]^ |
| 12 | TTF-COF | 2440 | 7.5×10^5^ | Pd(II), Cd(II), Ni(II), Zn(II), Na(I), Mg(II), K(I), Cr(III), Cu(II), Fe(III) | 6 | ^[^^27^^]^ |
| 13 | PE/PP-g-PDMAEMA | 949 | 2.1×10^4^ | Fe(III), Ni(II), Pb(II), Cu(II) | 5 | ^[^^28^^]^ |
| 14 | BUT-33-PpPD | 1600 | 6.7×10^4^ | Na(I), K(I), Mg(II), Ca(II), Ni(II), Cu(II), Zn(II) | 5 | ^[^^29^^]^ |
| 15 | PAF-147/polydopamine | 1700 | 2.0×10^3^ | Na(I), Mg(II), Ca(II), K(I), Ni(II), Cu(II), Zn(II) | 6 | ^[^^30^^]^ |
| 16 | CS-ES | 657 | 1.0×10^5^ | Ni(II), Cu(II), Co(II), Mg(II), Zn(II), Ca(II) | 4 | ^[^^31^^]^ |
| 17 | BCS | 1072 | 1.5×10^2^ | Li(I), Cd(II), Mg(II), Ni(II), Zn(II) | 3 | ^[^^32^^]^ |
| 18 | CS-MoS_2_-3 | 3109 | 7.5×10^3^ | Cu(II), Mg(II), Ni(II), Li(I), Zn(II) | 4 | ^[^^33^^]^ |
| 19 | Cu_31_S_16_ | 6274 | 2.5×10^7^ | Na(I), K(I), Ca(II), Mg(II), Cd(II), Pb(II), Cr(III), Mn(II), Fe(III), Ni(II), Co(II), Zn(II), Cu(II) | 5 | This work |

**Abbreviations:** K_d_ quantifies the partitioning of Au(III) between the solid and liquid phases. CMTU, Cu_2_O and thiourea functionalized MCM-41; Cu_2_O-TU, thiourea-modified Cu_2_O; MPVA, 3D Ti_3_C_2_ MXene-POSS composite aerogel; Fe-BTC/pTA, metal-organic framework/polymer composite; JNM-103, imine-linked Cu-CTU CMOFs (JNM stands for Jinan materials) ; JNM-100-AO, vinylene-linked Ag(I) CTU-based MOFs (JNM stands for Jinan materials); Ionic-COF-Cl, crystalline ionic COF (synthesized from 1,2,3-triaminoguanidine hydrochloride (TG-Cl) and 4′,4′′,4′′-(1,3,5-triazine-2,4,6-triayl) tris ([1,1′-biphenyl]-4-carbaldehide) (TFBT)); TpDa-COF, acridine-based covalent organic framework; COF-HNU-25, Schiff-based COFs containing high-density H-bond nanostraps; IPcomp-9, crystalline ionic hybrid aerogel material (IP stands for IISER Pune, comp stands for composite); TTF-COF, vinyl-linked covalent organic frameworks (VCOFs) incorporating TTF and tetraphenylethylene (TPE) units; PE/PP-g-PDMAEMA, N,N-Dimethylamino ethyl methacrylate (DMAEMA) grafted on polyethylene-coated polypropylene skin-core structure fiber (PE/PP); BUT-33-PpPD, Ni(II)-pyrazolate (pz) MOF, Beijing University of Technology (BUT)-33, supported with poly(para-phenylenediamine) (PpPD); PAF-147/polydopamine, porous aromatic framework (PAF)/polymer composite; CS-ES, chitosan modified with poly(ethylene sulfide); BCS, benzothiazole modified chitosan; CS-MoS_2_-3, MoS_2_ integrated into cross-linking chitosan; GCC51, glutaraldehyde-crosslinked chitosan beads.

**Supplementary Table 7.** Comparison of chemical consumption and costs for gold recovery from 1.0 t of CPU scraps using three methods.

| Method | Chemical | Consumed mass  (kg) | Unit price  ($ kg^-1^) | Subtotal  ($) |
| --- | --- | --- | --- | --- |
| Chemical precipitation ^a^ | NMP | 5000 | 1.520 | 7600.00 |
|  | HNO_3_ | 51 | 0.022 | 1.12 |
|  | NaOH | 25.3 | 0.348 | 8.80 |
| Electrochemical ^b^ | Na_2_S_2_O_5_ | 0.91 | 0.490 | 0.45 |
|  | Electrolyte | 3.12 | 5.970 | 18.63 |
|  | HCl | 475.97 | 0.022 | 10.47 |
| This work | Cu_2_O | 23.608 | 0.383 | 9.04 |
|  | NaBH_4_ | 259.463 | 0.0202 | 5.24 |
|  | Na_2_S | 3594.015 | 0.102 | 366.59 |
|  | HCl | 0.022 | 24.22 | 0.53 |
|  | HNO_3_ | 0.21 | 21.79 | 4.58 |

Note: ^a^ The data for the chemical precipitation method were calculated according to the report by Soares et al.,^[^^34^^]^ who used *N*-methyl-2-pyrrolidone (NMP) to extract gold from CPU scraps. Recovery and reuse of the precipitant were not assumed in the calculation.

^b^ The data for the electrochemical method were calculated according to the report by Lister et al.,^[^^35^^]^ who used a thiosulfate-based leaching process for gold extraction.

**Supplementary Table 8.** Comparison of various consumptions for Au recovery from 1.0 t of CPU scraps using three methods.

| Metrics | This work | Chemical  precipitation ^c^ | Electrochemical ^c^ |
| --- | --- | --- | --- |
| Chemical consumption (kg) | 46.52 | 5076.3 | 480 |
| Water consumption (kg) ^a^ | 186.8 | 206 | 835 |
| Electricity consumption (kWh) ^b^ | 356.167 | 5672 | 7187.28 |
| Selectivity coefficient (%) | 99.6 | 27 | 73 |
| Recovery efficiency (%) | 98.8 | 74 | 85.3 |

Note: ^a^ Water consumption refers to the total volume of water used throughout the process of gold recovery from 1.0 ton of CPU scraps. For the electrochemical route, the relatively high water demand primarily arises from leachant and electrolyte preparation, solution dilution, product and electrode washing, and solution replacement during process operation.^[^^35^^]^

^b^ Energy consumption refers to the amount of energy required to recover gold from 1.0 ton of dissolved CPU scraps.

^c^ The data for the chemical precipitation method and the electrochemical method were calculated according to the report by Chen et al.^[^^36^^]^

**Supplementary Table 9.** Comparison of Material and electricity costs for Au recovery from 1.0 t of CPU scraps using three methods.

| Metrics | | This work | Chemical  precipitation | Electrochemical |
| --- | --- | --- | --- | --- |
| Cost ($) | Chemical | 385.98 | 7609.92 | 29.55 |
|  | CPU | 13717.00 | 13717.00 | 13717.00 |
|  | Water | 15.88 | 17.51 | 70.98 |
|  | Electrical | 21.37 | 340.32 | 431.24 |
|  | Total | 14140.23 | 21684.75 | 14248.76 |
| Recovery value ($) | | 533473.50 | 399970.00 | 461046.50 |

**References**

[1]	X. Zhang, Y. Guo, J. Tian, B. Sun, Z. Liang, X. Xu, H. Cui, *Applied Catalysis B: Environmental* **2018**, *232*, 355, https://doi.org/10.1016/j.apcatb.2018.03.074.

[2]	D. Mei, B. Yan, *Small* **2023**, *19* (48), 2304811, https://doi.org/10.1002/smll.202304811.

[3]	M. Monier, D. A. Abdel-Latif, H. A. Mohammed, *International Journal of Biological Macromolecules* **2015**, *75*, 354, https://doi.org/10.1016/j.ijbiomac.2014.12.001.

[4]	C. Liu, Z. Song, C. Wang, P. Lan, Y. Yang, X. Yan, H. Kang, M. Ma, D. Gu, Y. Liu, X. Yan, Y. Mao, *Journal of Solid State Chemistry* **2023**, *319*, 123813, https://doi.org/10.1016/j.jssc.2022.123813.

[5]	E. Oguz, *Arabian Journal of Chemistry* *10*, https://doi.org/10.1016/j.arabjc.2014.10.008.

[6]	M. Mann, T. P. Nicholls, H. D. Patel, L. S. Lisboa, J. M. M. Pople, L. N. Pham, M. J. H. Worthington, M. R. Smith, Y. Yin, G. G. Andersson, C. T. Gibson, L. J. Esdaile, C. E. Lenehan, M. L. Coote, Z. Jia, J. M. Chalker, *Nature Sustainability* **2025**, *8* (8), 947, https://doi.org/10.1038/s41893-025-01586-w.

[7]	R. Li, S. Yan, T. Xue, R. Qiu, Y. Li, W. Hao, G. Xu, Y. Wang, Y. Hong, Y. Su, H. Wang, S. Yang, L. Peng, J. Li, *Nano Research* **2024**, *17* (1), 382, https://doi.org/10.1007/s12274-023-6077-0.

[8]	K. Fu, X. Liu, X. Zhang, S. Zhou, N. Zhu, Y. Pei, J. Luo, *Nature Communications* **2024**, *15* (1), 6137, https://doi.org/10.1038/s41467-024-50595-4.

[9]	D. Danaci, P. A. Webley, C. Petit, *Frontiers in Chemical Engineering* **2021**, *Volume 2 - 2020*, https://doi.org/10.3389/fceng.2020.602430.

[10]	F. Li, J. Zhu, P. Sun, M. Zhang, Z. Li, D. Xu, X. Gong, X. Zou, A. K. Geim, Y. Su, H.-M. Cheng, *Nature Communications* **2022**, *13* (1), 4472, https://doi.org/10.1038/s41467-022-32204-4.

[11]	J. Wang, K. Jia, J. Ma, Z. Liang, Z. Zhuang, Y. Zhao, B. Li, G. Zhou, H.-M. Cheng, *Nature Sustainability* **2023**, *6* (7), 797, https://doi.org/10.1038/s41893-023-01094-9.

[12]	M. Arshadi, S. Yaghmaei, S. M. Mousavi, *Resources, Conservation and Recycling* **2018**, *139*, 298, https://doi.org/10.1016/j.resconrec.2018.08.013.

[13]	T. Huang, J. Zhu, X. Huang, J. Ruan, Z. Xu, *Waste Management* **2022**, *139*, 105, https://doi.org/10.1016/j.wasman.2021.12.030.

[14]	Z. M. Yunus, A. Al-Gheethi, N. Othman, R. Hamdan, N. N. Ruslan, *Journal of Cleaner Production* **2020**, *251*, 119738, https://doi.org/10.1016/j.jclepro.2019.119738.

[15]	M. M. Wright, D. E. Daugaard, J. A. Satrio, R. C. Brown, *Fuel* **2010**, *89*, S2, https://doi.org/10.1016/j.fuel.2010.07.029.

[16]	S. E. A. Elhafez, H. A. Hamad, A. A. Zaatout, G. F. Malash, *Environmental Science and Pollution Research* **2017**, *24* (2), 1397, https://doi.org/10.1007/s11356-016-7891-7.

[17]	C. Liu, Y. Mao, Y. Yang, Z. Song, C. Wang, H. Kang, X. Yan, D. Gu, X. Yan, Z. Wang, *Chemical Engineering Journal* **2022**, *448*, 137651, https://doi.org/10.1016/j.cej.2022.137651.

[18]	C. Liu, Y. Mao, Z. Song, Y. Yang, X. Yan, H. Kang, Y. Liu, D. Gu, X. Zhang, X. Yan, *Journal of Environmental Chemical Engineering* **2022**, *10* (4), 108162, https://doi.org/10.1016/j.jece.2022.108162.

[19]	W. Wang, X. Li, R. Wang, J. Wang, H. Deng, S. Tong, *Chemical Engineering Journal* **2023**, *468*, 143453, https://doi.org/10.1016/j.cej.2023.143453.

[20]	J. Luo, J.-T. Lin, X. Luo, N. Yin, M. Xie, X. Chen, Y.-Q. Li, Y.-G. Li, G.-H. Ning, D. Li, *Angewandte Chemie International Edition* **2025**, *64* (27), e202502749, https://doi.org/10.1002/anie.202502749.

[21]	J. Luo, X. Luo, M. Xie, H.-Z. Li, H. Duan, H.-G. Zhou, R.-J. Wei, G.-H. Ning, D. Li, *Nature Communications* **2022**, *13* (1), 7771, https://doi.org/10.1038/s41467-022-35467-z.

[22]	C. Liu, Z. Wang, S. Zhang, Y. Xiang, Z. Li, L. Xie, H. Zeng, *Chemical Engineering Journal* **2025**, *505*, 159231, https://doi.org/10.1016/j.cej.2025.159231.

[23]	J. Zhao, Z. Qiao, Y. He, R. Zhang, H. Li, X. Song, D. Cao, S. Wang, *Angewandte Chemie International Edition* **2025**, *64* (2), e202414366, https://doi.org/10.1002/anie.202414366.

[24]	J. Qiu, C. Xu, X. Xu, Y. Zhao, Y. Zhao, Y. Zhao, J. Wang, *Angewandte Chemie International Edition* **2023**, *62* (17), e202300459, https://doi.org/10.1002/anie.202300459.

[25]	D. Mei, B. Yan, *Angewandte Chemie International Edition* **2024**, *63* (24), e202402205, https://doi.org/10.1002/anie.202402205.

[26]	D. Majumder, S. Fajal, M. M. Shirolkar, A. Torris, Y. Banyla, K. Biswas, S. Rasaily, S. K. Ghosh, *Angewandte Chemie International Edition* **2025**, *64* (7), e202419830, https://doi.org/10.1002/anie.202419830.

[27]	A. Zadehnazari, F. Auras, A. A. Altaf, A. Zarei, A. Khosropour, S. Amirjalayer, A. Abbaspourrad, *Nature Communications* **2024**, *15* (1), 10846, https://doi.org/10.1038/s41467-024-55156-3.

[28]	X. Liu, J. Ao, X. Yang, C. Ling, B. Zhang, Z. Wang, M. Yu, R. Shen, H. Ma, J. Li, *Journal of Applied Polymer Science* **2017**, *134* (25), https://doi.org/10.1002/app.44955.

[29]	T. Xue, T. He, L. Peng, O. A. Syzgantseva, R. Li, C. Liu, D. T. Sun, G. Xu, R. Qiu, Y. Wang, S. Yang, J. Li, J.-R. Li, W. L. Queen, *Science Advances* *9* (13), eadg4923, https://doi.org/10.1126/sciadv.adg4923.

[30]	T. Xue, G. Xu, C. Liu, R. Li, Y. Wu, Y. Guo, S. Gong, Z. Shao, X. Cai, H. Zou, L. Cui, J. Zhao, Z. Gao, S. Yang, J. Li, B. Han, L. Peng, *Angewandte Chemie International Edition* **2025**, *64* (18), e202500092, https://doi.org/10.1002/anie.202500092.

[31]	S. Wang, H. Wang, S. Wang, L. Zhang, L. Fu, *Microporous and Mesoporous Materials* **2022**, *341*, 112074, https://doi.org/10.1016/j.micromeso.2022.112074.

[32]	L. Chen, J. Tang, X. Zhang, S. Wang, Z. Ren, *International Journal of Biological Macromolecules* **2021**, *193*, 1918, https://doi.org/10.1016/j.ijbiomac.2021.11.023.

[33]	M. Zhao, Z. Huang, S. Wang, L. Zhang, *Chemical Engineering Journal* **2020**, *401*, 126006, https://doi.org/10.1016/j.cej.2020.126006.

[34]	P. M. S. Sousa, L. M. Martelo, A. T. Marques, M. M.S.M. Bastos, H. M.V.M. Soares, *Chemical Engineering Journal* **2022**, *434*, 134604, https://doi.org/10.1016/j.cej.2022.134604.

[35]	L. A. Diaz, T. E. Lister, *Waste Management* **2018**, *74*, 384, https://doi.org/10.1016/j.wasman.2017.11.050.

[36]	X. Chen, Q.-Z. Zhong, Z. Qian, L. B. T. Nguyen, J. R. T. Chen, E. X. Tan, K. Wang, F. Song, J. J. Richardson, Y. Lv, X. Y. Ling, T. Liu, *Nature Water* **2026**, *4* (3), 360, https://doi.org/10.1038/s44221-026-00591-3.
